# Is chimerism associated with cancer across the tree of life?

**DOI:** 10.1371/journal.pone.0287901

**Published:** 2023-06-29

**Authors:** Stefania E. Kapsetaki, Angelo Fortunato, Zachary Compton, Shawn M. Rupp, Zaid Nour, Skyelyn Riggs-Davis, Dylan Stephenson, Elizabeth G. Duke, Amy M. Boddy, Tara M. Harrison, Carlo C. Maley, Athena Aktipis

**Affiliations:** 1 Arizona Cancer Evolution Center, Arizona State University, Tempe, AZ, United States of America; 2 Biodesign Institute, Center for Biocomputing, Security and Society, Arizona State University, Tempe, AZ, United States of America; 3 School of Life Sciences, Arizona State University, Tempe, AZ, United States of America; 4 Department of Psychology, Arizona State University, Tempe, AZ, United States of America; 5 Department of Clinical Sciences, North Carolina State University, Raleigh, NC, United States of America; 6 Exotic Species Cancer Research Alliance, North Carolina State University, Raleigh, NC, United States of America; 7 Department of Anthropology, University of California, Santa Barbara, CA, United States of America; Laboratoire de Biologie du Développement de Villefranche-sur-Mer, FRANCE

## Abstract

Chimerism is a widespread phenomenon across the tree of life. It is defined as a multicellular organism composed of cells from other genetically distinct entities. This ability to ‘tolerate’ non-self cells may be linked to susceptibility to diseases like cancer. Here we test whether chimerism is associated with cancers across obligately multicellular organisms in the tree of life. We classified 12 obligately multicellular taxa from lowest to highest chimerism levels based on the existing literature on the presence of chimerism in these species. We then tested for associations of chimerism with tumour invasiveness, neoplasia (benign or malignant) prevalence and malignancy prevalence in 11 terrestrial mammalian species. We found that taxa with higher levels of chimerism have higher tumour invasiveness, though there was no association between malignancy or neoplasia and chimerism among mammals. This suggests that there may be an important biological relationship between chimerism and susceptibility to tissue invasion by cancerous cells. Studying chimerism might help us identify mechanisms underlying invasive cancers and also could provide insights into the detection and management of emerging transmissible cancers.

## Introduction

### Chimerism is widespread across life

Although the concept of a chimera derives from a Greek mythological monster with a lion’s head, a goat’s body, and a snake’s tail [1,2], chimeras are biologically real. A chimera is an obligately multicellular organism that is composed of non-clonal cells (relatedness < 1), which do not originate from mutations within the body [3–5]. Chimeric cells are cells from a different host. This can range from large scale cellular exchange to smaller amounts, called microchimerism. Chimeras exist in several taxa, from marine sponges to trees and terrestrial mammals (Tables 1 and 2).

**Table 1:** Examples of natural (A) and experimental (B) chimeras across 12 obligately multicellular taxa.

**A.**						
**Taxon (common name)**	**Examples of chimerism**	**Chimerism early in development**	**Chimerism later in development**	**Manipulation of the graft/recipient**	**For how long did the graft cells survive in the recipient?**	**How many of the graft cells survived in the recipient?**
Vertebrata (vertebrates)	see Table 2A	**✓** (see Table 2A)	**✓** (see Table 2A)	see Table 2A	see Table 2A	see Table 2A
Tunicata (tunicates)	chimeric colonies of 2–3 different genotypes [6]	N/A	N/A	NA	N/A	N/A
Cnidaria (cnidarians)	chimeras with distinct genotypes, unrelated genotypes fused [7]	**✓** juveniles [7–9]	**✓** [7–9]	“branches were sampled as far away from each other as possible” [7,9]	up to 450 days [8]	“partners shared layers of endoderm, mesoglea and ectoderm, and the gastrovascular cavity” [10]
Porifera (sponges)	bispecific conglomerates [11]	**✓** larvae [12]	**✓** [12]	N/A	N/A	10% of the whole [11]
Basidiomycota (filamentous fungi)	single basidiome with nine different genotypes [13]	N/A	N/A	N/A	N/A	N/A
**B.**						
**Taxon (common name)**	**Examples of chimerism**	**Chimerism early in development**	**Chimerism later in development**	**Manipulation of the graft/recipient**	**For how long did the graft cells survive in the recipient?**	**How many of the graft cells survived in the recipient?**
Vertebrata (vertebrates)	see Table 2B	**✓** (see Table 2B)	**✓** (see Table 2B)	see Table 2B	see Table 2B	see Table 2B
Tunicata (tunicates)	fusing colonies share at least one allele [14,15]; xenograft chimera [16]	**✓** [17]	**✓** adult [17] sexually mature [15]	irradiated subclones received the grafts [6,16]	30 days [6]; 1 week [18]; months [16]; 2 months [14]; 8–10 months [15]	both genotypes in a bud [14]; mass of gonads and soma came from the resorbed genotype [15]
Protostomia (protostomes)	allografts [19]; allografts and xenografts [20]; xenografts [21]	N/A	**✓** adults [21]; parents [22]	collagen-based skin-like scaffold allowed wound healing [19]	6 months [19]; 6 months [20]; 14 days [22]	implanted hearts continued to beat throughout the study [19]
Placozoa (placozoans)	cell reaggregation, no evidence that the new organism is functional [23]	N/A	N/A	dissociation medium, cells were passed through a gauze [23]	weeks [23]	N/A
Ctenophora (comb jellies)	“animal consisting of the mid-pieces of four animals” [24]	N/A	N/A	grafts were held in place with cotton to permit healing [25]	3 days [24]; 2 days, 10 days [25]	“each piece maintained its identity”, “and by regenerating auricles and lobes” [24]
Echinodermata (echinoderms)	viable allografts [26]	N/A	**✓** adults [26]	anesthetized before grafting, use of tetracycline and gelatin powder [26]	>100 days, 129–185 days [26]; >300 days, 110 days [27]; average of 341.8 days [28]	allografts “gradually resorbed by ingrowth of recipient tissue” [27]
Cnidaria (cnidarians)	bispecific chimeras [29]	N/A	N/A	N/A	>4 months [29]	“chimeras had *H*. *attenuata* epithelial cells and *P*. *oligactis* interstitial cell lineage” [29]
Porifera (sponges)	bispecific conglomerates [30]	**✓** larvae [31,32]	N/A	“forced to settle in contact” [32]	48 hours, 72 hours [33]; 50 days [32]	N/A
Ascomycota (sac fungi)	hyphae fuse between colonies of the same species [34]	**✓** germinating conidia [35]	**✓** mature hyphae [35]	“conidia were used to initiate heterokaryotic mycelia.” [35]	N/A	N/A
Embryophyta (land plants)	interspecific chimeras [36–38]	**✓** [39]; seedlings [40]; young plants [38]	N/A	“Graft unions were secured with budding rubbers” [40]	46, 53, 70 and 73 days after decapitation [37]	“Eventually the *N*. *glauca* tissue in the original menstem was replaced by *N*. *tabacum*” [37]
Rhodophyta (red algae)	“interindividual fusions in red and brown algal species” [41]	**✓** [41]	N/A	laboratory-built bicolor chimeras [41]	30–45 days [41]	“mixed tissue reached 10%–15% the axes length” [41]

In the majority of cases in the literature, species reject foreign cells. The list of references in this table is not exhaustive since we do not mention here all the examples of graft rejection reported in the literature. The examples of chimerism in this table are the examples of graft/foreign cell acceptance from the existing literature. We provide an extended version of this table, which also includes the highest level of chimerism observed in each taxon, in S1 Table.

**Table 2:** Examples of natural (A) and experimental (B) chimerism in 18 mammalian species.

**A.**						
**Mammalian species (common name)**		**Examples of chimerism**	**Chimerism early in development**	**Chimerism later in development**	**For how long did the graft cells survive in the recipient?**	**How many of the graft cells survived in the recipient?**
*Saguinus oedipus* (cotton-top tamarin)		microchimerism [42]	**✓** [42]	N/A	N/A	chimeric lymphocyte and monocyte/macrophage populations [42]
*Bos taurus* (cattle)		microchimerism [43]	**✓** [43]	**✓** [43]	twins were adults when tested [43]	N/A
*Canis lupus familiaris* (domestic dog)		fetal microchimerism [44]	**✓** [44]	N/A	N/A	N/A
*Homo sapiens* (human)		blood group chimerism [45]	**✓** [45–48]	**✓** [46–48]	years, probably a lifetime [48]; until adulthood [46]	mothers with scleroderma disease had “an average of seven male cells per 10 milliliters of blood”, one fetal cell in 1 million cells in maternal circulation [46]
*Callithrix jacchus* (common marmoset)		blood chimerism between twins and triplets [49]	**✓** [49]	N/A	age at birth or age when euthanized [49]	N/A
*Equus ferus* (wild horse)		microchimerism [50]	**✓** [50]	N/A	6th month of gestation [50]	N/A
*Macaca mulatta* (rhesus macaque)		maternal microchimerism [51]	**✓** [51]	**✓** [51]	1–1.5 years of age [51]	0.001–1.9% chimeric cells [51]
**B.**					
**Mammalian species (common name)**	**Examples of chimerism**	**Chimerism early in development**	**Chimerism later in development**	**Manipulation of the graft/recipient**	**For how long did the graft cells survive in the recipient?**	**How many of the graft cells survived in the recipient?**
*Rattus norvegicus* (common rat)	allogeneic [52]; mouse retinae transplanted into rats [53]	**✓** [53,54]	N/A	conditioned with 1100 cGy and T cell depleted [52]	300 days after transplantation, ≥14 months [52]		“well-formed grafts containing numerous cells” [53]
*Mus musculus* (house mouse)	human embryonic cells implanted in mice [55]	**✓** [55–57]	N/A	coculturing with mouse embryonic fibroblasts in defined medium [55]	two months, 18 months [55]; 2 weeks, 3–5 weeks [57]	“0.1% of the brain cells are of human origin.” [55]
*Sarcophilus harrisii* (Tasmanian devil)	“All successful allografts were acutely rejected” [58]	N/A	**✓** [58]	surgical glue on the borders of grafts, pain relief medication [58]	“14 days after surgery”, “severe rejection on Day 21” [58]	“necrosis associated with polymorphonuclear cell infiltration” [58]
*Ovis aries* (sheep)	embryonically derived human hematopoietic stem cells in sheep [59,60]	**✓** [59,60]	N/A	“transplanted early in gestation when the recipient is still largely immunologically naive.” [60]	7 years later [59,60]; “at least 9 months.” [60]	“liver, heart and pancreas, that are 15% human.” [59,60]
*Bos taurus* (cattle)	transgene-specific sequence in cows with transgenic fetuses [61]	**✓** [61]	**✓** [61]	“embryo produced by in vitro fertilization of transvaginally recovered oocytes” [61,62]	up to 4 months [61]	“six circulating male cells or their corresponding DNA contents (if cell-free) per mL of maternal blood” [61]
*Sus scrofa* (wild boar)	human cells in pigs	**✓** [63]	N/A	fetal pigs injected with human T cell-depleted bone marrow cells [63]	>1 year [63]	N/A
*Acinonyx jubatus* (cheetah)	allograft, “skin grafts between unrelated cheetahs” [64]	N/A	**✓** [64]	antibiotics were administered, surgical area was bandaged [64]	“2 weeks after surgery”, day 23 [64]	N/A
*Canis lupus familiaris* (domestic dog)	allografts in dogs bearing identical DLA haplotypes [65]; skin allograft [66]	N/A	**✓** [65,66]	200 cGy, immunosuppression [66]; radiation chimeras [65]	4 weeks, 76 weeks, >5 years [66]; 10–25 days [65]	“T and B cells contained donor-type cells” [66]
*Papio hamadryas* (Hamadryas baboon)	xenotransplantation [67]; pig-to-baboon xenografts [68]	**✓** [68]	N/A	immunosuppressives, irradiation, aspirin [67]; fibrinogen [68]	up to 6 months, 78 and 179 days [67]; up to 3 ½ days [68]	N/A
*Homo sapiens* (human)	allogeneic transplantation [69]; xenograft [70]	N/A	**✓** [69,70]	immunosuppression, “20 units of blood were given during the 11 h operation.” [70]	70 days [70]	N/A
*Didelphis virginiana* (Virginia opossum)	maternal allograft, “none of the 24 young less than 12 days of age rejected the maternal allografts” [71]	**✓** [71]	**✓** [71]	“the mother (was) anesthetised with sodium pentobarbital” [71]	“at least 80 days in most cases” [71]	N/A
*Oryctolagus cuniculus* (European rabbit)	xenografted rat tissue [72]	N/A	N/A	N/A	“rat hippocampal grafts developing for 8 weeks in the rabbit septum” [72]	“grafts significantly increased in their volume (600 to 800% of the initial value)” [72]
*Dasypus novemcinctus* (armadillo)	skin grafts accepted between monozygotic littermates [73]	**✓** [73]	N/A	“grafts were redressed at the early inspections to protect them from trauma.” [73]	20 days, 50 days, 85th postoperative day [73]	N/A
*Mesocricetus auratus* (Syrian hamster)	homografts between completely unrelated stocks [74]	N/A	**✓** [74]	“graft fitted into an appropriately sized bed on the lateral thoracic wall.” [74]	200 days, 24 to 140 days [74]	N/A

In the majority of cases in the literature, species reject foreign cells. The list of references in the table is not exhaustive since we do not mention here all the examples of graft rejection reported in the literature. The examples of chimerism in this table are examples of graft/foreign cell acceptance from the existing literature. An extended version with the highest level of chimerism observed in each species is reported in S2 Table. cGY: Centigray; DLA: Dog leucocyte antigen.

The evolutionary road from single cells to obligate multicellularity, shows that irreversible (obligate) multicellularity only arose when cells divided clonally across the tree of life [75,76]. Such clonality allowed somatic cells to cooperate at the most extreme level, altruistically sacrificing their reproduction, due to the alignment of their genomic fitness interests with the germ cells and other somatic cells. Chimeric cells, due to their non-clonality, are expected to have incompletely aligned fitness interests with other cells in the obligately multicellular body [75–77]. These unaligned fitness interests and other interacting ecological factors can lead to conflict among cells [75,76,78–81], which can include overproliferation, avoiding apoptosis and other forms of cheating among cells that can lead to fatal cancers [77,82,83]. For example, detection of Y chromosomes in the mother (male microchimerism) has been associated with a higher risk of developing colon cancer [84]. However, male microchimerism has also been associated with a decreased risk of developing breast cancer [84]. When non-self cells appear, self/non-self-recognising systems, often in the form of immune cells, destroy the foreign/non-relatives [85,86]. This is why, in organ transplantations, it is necessary to give immunosuppressive drugs to the host, otherwise, the immune system almost always rejects the transplant [87–89].

The existence of chimeras challenges the traditional view that clonality is critical to obligate multicellularity [75–77]. Chimeras also appear to break the traditional laws of inheritance [90]. Mendel saw that traits can be inherited strictly through the passage of parental traits in the germline creating the zygote [91]. Since then, people have discovered that somatic cells are routinely transmitted from one generation to the next via the placenta, generating microchimerism [46,47,92]. Transplanted cells can also become *the* germline, as was seen in a patient who received a bone marrow transplant where after four years, 100% of his semen DNA derived from the donor [93]. Similarly, in the colonial tunicate *Botryllus schlosseri*, when different colonies fuse forming a chimera, the germ cells of one colony can replace the germ cells of another colony, a phenomenon known as germ cell parasitism [94–96].

### Obligate chimerism and cancer have common hallmarks

Cancers are composed of genetically and/or epigenetically mutated cells that invade other tissues and/or organs in the body [97–99]. Cancer cells can even invade other hosts, such as humans [100–103], Tasmanian devils, Syrian hamsters, dogs, clams, and molluscs as intraspecies or interspecies transmissible cancers [104–110]. Across different types of cancers we see similar phenotypes called hallmarks of cancer [111]. Cancers and chimerism share some of these hallmarks. Cancer cells can evade immune destruction [112–115]; chimeric cells can too [47,116]. For instance, transmissible cancers are linked with loss of diversity in the major histocompatibility complex (MHC), low expression of MHC antigens, in other words immunological invisibility, and appear to be boundless in terms of the intraspecific hosts they can invade (e.g., 4,000–8,500 year old distribution of Canine Transmissible Venereal Tumours across 43 countries) [117]. The membrane protein CD200 inhibits natural killer cell responses to cancer, facilitates graft tolerance in humans and mice, and is highly expressed on transmissible devil facial tumour cells [118]. Also, both cancer cells and chimeric endothelial progenitor fetal cells can induce angiogenesis and activate invasion [47,111,119–122]. Chimeric fetal cells have been found in several tumours in mothers [123–126]. Furthermore, chimeric fetal cells are more often found in cancerous than healthy tissue [127–129]. Genetic differences among cells can lead to conflict over limited resources, and overproliferation of one cell type over the other [77]. This may be happening when allografts or xenografts lead to hyperplasias in sea cucumbers, sea stars, and sponges [26,130,131], and interspecific crosses can lead to cancer in plants [111,132].

### Investigating associations between chimerism levels and cancer across species

It is clear that chimerism exists, but it has not been systematically studied in relation to susceptibility to cancer. In this study we use literature resources and zoological data across the tree of life to: (1) organise taxa according to their highest level of chimerism observed; and (2) test whether there is a positive association between the highest level of chimerism observed in an obligately multicellular taxon and the highest level of tumour invasiveness observed in those taxa. Within mammals, we also investigated whether chimerism was associated with neoplasia (benign or malignant) prevalence and malignancy prevalence. Due to the limited experimental data on tumour invasiveness in non-vertebrates in the literature, we classified the data relative to lineages representing broad taxa (from subphyla to subkingdoms) including: Vertebrata (vertebrates), Tunicata (tunicates), Protostomia (protostomes), Placozoa (placozoans), Ctenophora (comb jellies), Porifera (sponges), Echinodermata (echinoderms), Cnidaria (cnidarians), Porifera (sponges), Ascomycota (sac fungi), Embryophyta (land plants), Rhodophyta (red algae). We chose these taxa based on an existing phylogeny of tumour invasiveness across taxa [111]. We hypothesised that chimerism is positively associated with cancers across the tree of life.

## Results

### Tumour invasiveness is positively correlated with chimerism across the tree of life

**Fig 1:**
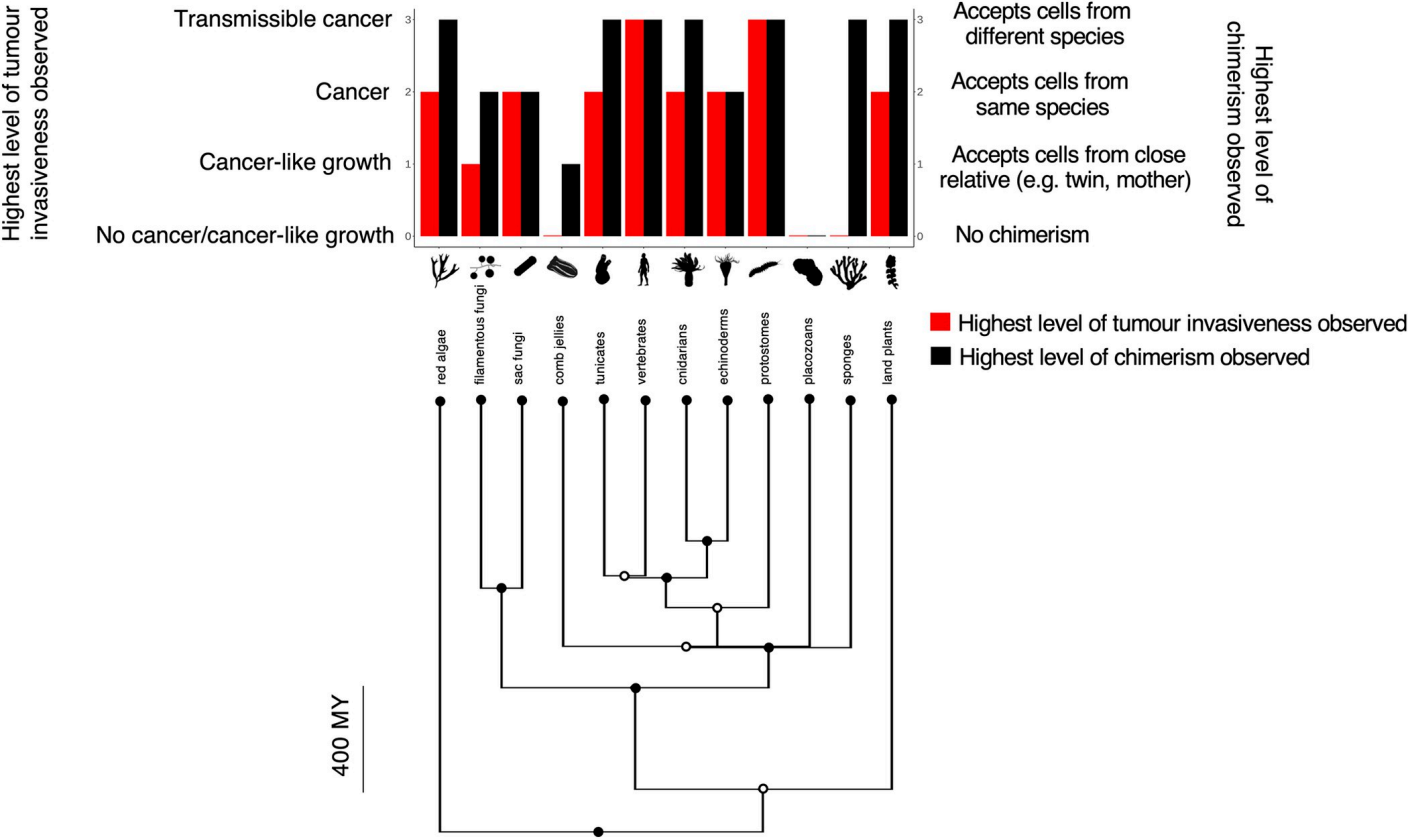
Phylogenetic tree of the highest level of chimerism observed and the highest level of tumour invasiveness observed in each of 12 obligately multicellular taxa across the tree of life. Red bars show the highest level of tumour invasiveness observed in each taxon, from low to high tumour invasiveness (no cancer observed, cancer/cancer-like phenomena, cancer, transmissible cancer). Black bars show the highest chimerism level observed in each taxon, from low to high chimerism (no chimerism observed, accepts cells from a close relative e.g., mother or twin, accepts cells from the same species other than a close relative, accepts cells from different species). The phylogenetic tree was created using the Time Tree of Life (http://timetree.org/). Images show example species in each taxon (images from http://phylopic.org/). The time bar shows millions of years (MY).

**Fig 2:**
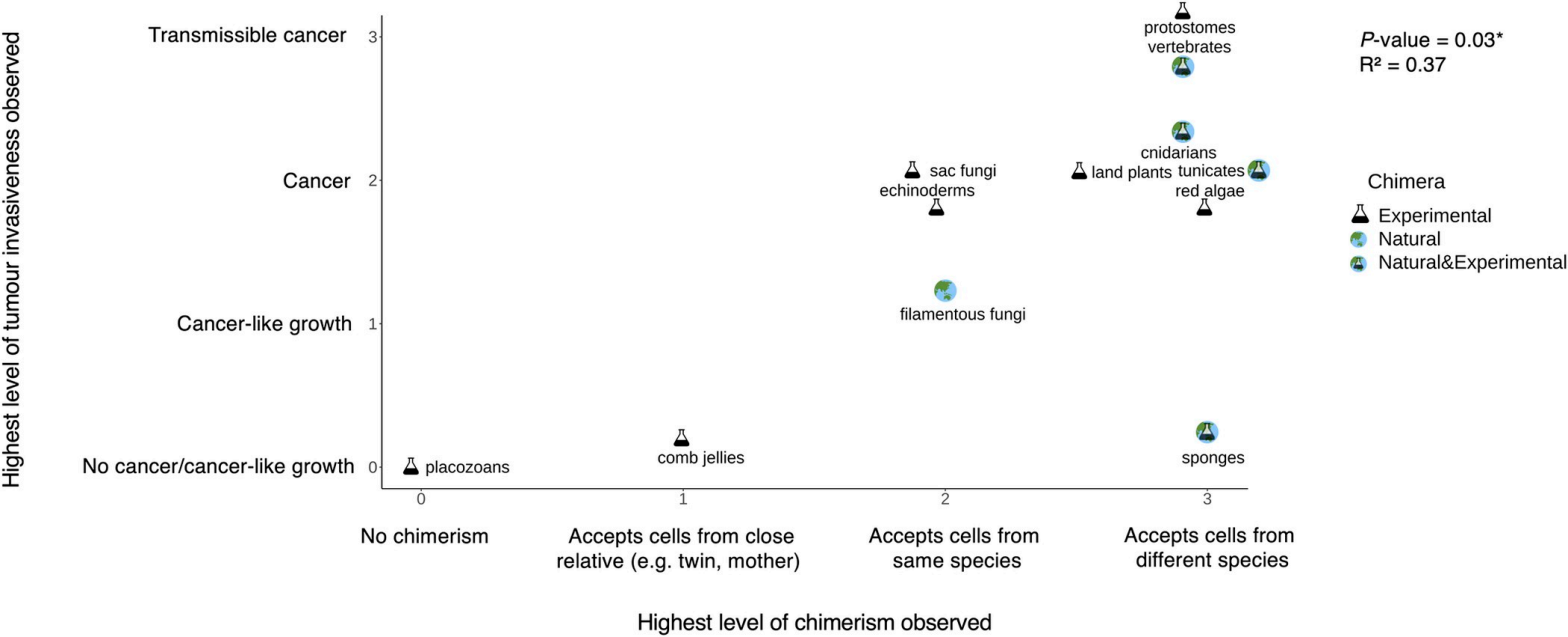
The highest level of tumour invasiveness observed is positively correlated with the highest level of chimerism observed across 12 obligately multicellular taxa on the tree of life (PGLS analysis: * *P*-value < 0.05). We show each taxon with a flask, a globe, or both, according to whether it consists of experimental chimeras (flask), natural chimeras (globe), or both. We use minimal jitter to better visualise individual taxa.

We classified tumour invasiveness on a scale from *no cancer or no cancer-like growth*, *cancer-like growth*, *cancer*, to *transmissible cancers*. We found that across 12 obligately multicellular taxa, the highest level of tumour invasiveness observed in a taxon is positively correlated with the highest level of chimerism observed in a taxon. In other words, in obligately multicellular taxa that accept foreign cells from different species, higher tumour invasiveness will be observed (Table 1; Figs 1 and 2; PGLS analysis: F-statistic = 6.02 on 1 and 10 DF, ML lambda = 1, R^2^ = 0.37, slope = 0.67, *P*-value = 0.03).

#### Types and duration of chimeras

Acceptance of foreign cells has been seen either as foreign cells existing inside species naturally, or species not rejecting graft tissue, or both (Tables 1 and S1). We have classified chimeras in these 12 obligately multicellular taxa as natural, experimentally-induced, or both (Tables 1 and S1; Fig 2). Seven taxa have been found to tolerate experimentally-induced chimeras. Only Basidiomycota exhibit evidence of just natural chimeras, and four taxa show evidence of both experimentally-induced and natural chimeras (Tables 1 and S1; Fig 2).

The length of time graft cells can survive in hosts varies from a few days to a lifetime, depending on the taxon. Graft cells have been found in many sites including the gonads, intestinal tissue, and meristem in plants (Tables 1 and S1). Chimerism has been reported early in development in two taxa, later in development in two taxa, both early and late in development in five taxa, and no information about developmental timing of chimerism is reported in three taxa (Table 1).

### Chimerism levels in terrestrial vs. aquatic taxa

Among the examined 12 obligately multicellular taxa, there was no significant correlation between chimerism levels and species being terrestrial vs. aquatic. In other words, obligately multicellular lineages that evolved on land did not have significantly different levels of chimerism than obligately multicellular taxa that evolved in water (S1 Fig).

### Chimerism does not explain the variance in malignancy and neoplasia prevalence across terrestrial mammals

**Fig 3:**
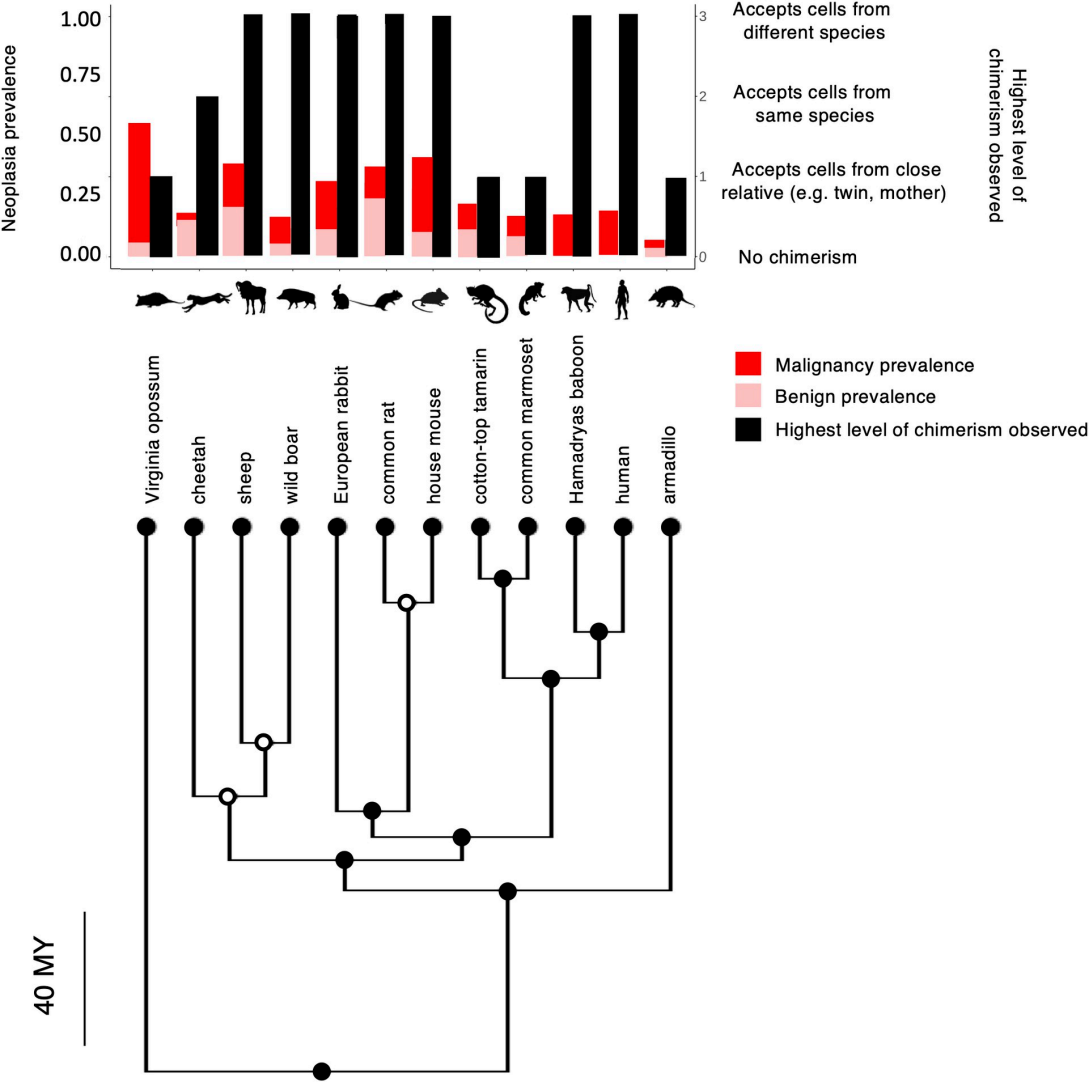
Phylogenetic tree of malignancy, neoplasia prevalence, and the highest levels of chimerism observed in 12 terrestrial mammalian species. Red shows the malignancy prevalence of each species. Pink shows the benign neoplasm prevalence of each species. Red together with pink show total neoplasia prevalence. In the case of adult humans, we only show their malignancy prevalence from https://ourworldindata.org, as we do not have data on their benign neoplasm prevalence. Black bars show the highest chimerism level observed in each species, from low to high chimerism (no chimerism observed, accepts cells from a close relative e.g., mother or twin, accepts cells from the same species other than close a relative, accepts cells from different species). The phylogenetic tree was created using the Time Tree of Life (http://timetree.org/). We obtained images of species from http://phylopic.org/. Time bar shows millions of years (MY).

We searched within mammalian species to see whether malignancy prevalence and neoplasia prevalence are correlated with chimerism levels. We found that the highest chimerism level observed does not explain a significant amount of variance in malignancy prevalence or neoplasia prevalence (Fig 3; PGLS analysis; malignancy prevalence: F-statistic = 0.34 on 2 and 8 DF, ML lambda = 0.00006, R^2^ = 0.2, *P*-value = 0.71; neoplasia prevalence: F-statistic = 0.48 on 2 and 8 DF, ML lambda = 0.99, R^2^ = 0.19, *P*-value = 0.63). The majority of these mammalian chimeras are developmentally young, immunosuppressed and/or inbred. Across the literature, eleven species are experimentally-induced chimeras, four species are natural chimeras, and three species including humans are both experimentally-induced and natural chimeras (Tables 2 and S2).

The period of time that the foreign cells survive in the host varies from 179 days in the Hamadryas baboon to a lifetime in humans. Graft cells have been found in many locations in the host, including the brain, spleen, liver, heart, pancreas, blood, and the thymus, depending on the species (Tables 2 and S2).

Based on the current literature, chimerism has been reported early in development in nine mammalian species, later in development in three species, both early and late in development in five species, and no data on the developmental timing of chimerism were available in the rabbit *Oryctolagus cuniculus* (Table 2).

## Discussion

Chimerism has been observed across a wide variety of species and appears in a variety of forms (Tables 1 and 2). We found that higher levels of chimerism are positively correlated with tumour invasiveness across 12 obligately multicellular taxa on the tree of life (Figs 1 and 2; Tables 1 and S1). We did not find a significant association between chimerism and malignancy prevalence or neoplasia prevalence among terrestrial mammals (Fig 3).

This association of chimerism with tumour invasiveness might be a result of similar underlying mechanisms that allow chimeric and cancerous cells to flourish inside hosts. This is consistent with several observations in the literature including common clinical signs between chimeric and cancer cells [47,111], hybrid crosses between different species of plants leading to cancerous growth [132], and even cell ploidy levels positively correlating with tumour invasiveness [133,134].

### Ecology may influence chimerism

Chimerism may also depend on the environment. Recent research shows that lineages forming non-clonal multicellular groups are more common in terrestrial environments, whereas lineages forming clonal groups are more common in aquatic environments [78,135,136]. This suggests we might expect to find more cases of chimeric cells that disrupt the cohesiveness of the multicellular community of cooperating cells in terrestrial environments. However, we did not find that obligately multicellular taxa that evolved on land had higher levels of chimerism than obligately multicellular taxa that evolved in water (S1 Fig). This could be because we restricted the definition of chimerism to obligately multicellular organisms, whereas Fisher et al. [78] restricted the definition of chimerism, i.e. non-clonal group formation, to facultatively multicellular organisms.

### Limitations & future directions

There are several limitations in this study. A primary limitation is the lack of data. We were only able to perform correlations using 12 obligately multicellular taxa across the tree of life (Fig 1) and 11 out of the 12 terrestrial mammalian species shown in Fig 3 (excluding humans due to the absence of relevant malignancy and neoplasia prevalence data). These large taxa likely include species with different degrees of chimerism. Additionally, there is likely a sampling bias in the degree of chimerism that has been observed. We are more likely to have observed chimerism, and higher levels of chimerism in species that have been studied more and for a longer period per experiment, compared to species for which there have been few studies. Thus, our analysis is just a first, imprecise view of the relationship between chimerism and cancer. Future studies would benefit from testing for both spontaneous and experimentally induced chimerism with consistent methodologies, across many more species. Such studies would also benefit from collecting more necropsies in those species (ideally in wild animals) for more accurate estimates of cancer prevalence.

Not all obligate chimeras are strictly chimeric throughout their lifetime and this creates additional confusion in the definition of multicellular species. Some chimeras keep chimeric cells only for a few days or months, while others for a lifetime (Tables 1 and 2). Some of this observed variation may be due to methodological differences among experiments (Tables 1 and 2), but other aspects of this variation are likely the result of biological differences among species, including differences in immunological barriers [137,138]. The specific immunological barriers that determine whether a graft will be accepted and/or passed to the next generation are largely unknown across species. For example, only recently have scientists found that chimeric cells can invade the germline of humans [93]. This reminds us that an individual may not be as defined as we think [76,77,139–141]. The tips of a phylogenetic tree may be a single species or even a chimera of two or more species [29,142]. In other words, according to Ford Doolittle, “the history of life cannot properly be represented as a tree.” [143].

For some species and higher taxonomic groups in our dataset, we know that there are chimeras early and/or late in development (Tables 1 and 2). We have too few species and higher taxonomic groups, however, to perform a powerful statistical analysis comparing chimerism levels between these different stages of development. Early embryonic development, pregnancy, and old age are times when the immune system is relatively weak. Working in a period when humanity was in desperate need for transplants for the injured, during and after World War II, Nobel Prize winner Peter Medawar et al. showed that immunological individuality is “*a property that comes into being during the course of development*” [144]. “*The chick*, *before the eighteenth day of incubation*, *is almost indiscriminately hospitable*” to extrinsic agents, 29 among 188 chickens that received a graft from a different individual “*on the day of hatching or within a few days thereafter*” could keep it almost indefinitely, but there is “*progressive decay*, *with increasing age*, *of the power of an antigenic stimulus to confer tolerance*.” [145–148]. Therefore, we would expect species to have a higher susceptibility in receiving and accepting chimeric cells during those vulnerable times of early development, pregnancy, and old age. Future studies should determine which developmental stage individuals can accept related/foreign cells.

Finally, we lack information on the molecular mechanisms behind the association between chimerism and tumour invasiveness for any given species. A molecular *in vitro* and *in vivo* approach would be useful to detect and track chimeric cells over several generations [149], identify whether and when they become cancerous, determine if the same pathways are used to reject chimeric and cancerous cells, and test whether their degree of invasiveness depends on the level of chimerism.

### Conclusions

The results of this study are a promising first step in understanding the origins of chimerism and have implications for the discovery and study of transmissible cancers. We hypothesise that species that are the most accepting of chimeric cells are also the species most likely to harbour transmissible cancers. Since chimerism and cancer have common hallmarks, such as evading immune destruction [112–116], inducing angiogenesis, and activating invasion [47,111,119–122], by finding the mechanisms that chimeric cells use to invade other organisms we may find the mechanisms that transmissible cancer cells use to invade other organisms and thus design drugs to target those pathways.

## Methods

### Chimerism

To find chimeras across the tree of life, we searched the Web of Science, StarPlus, Google Scholar, JStor, and Mendeley using the keywords *transplantation*, *immune tolerance*, *immune development*, *microchimerism*, *genetic mosaicism*, *grafting*, *chimerism*, *chimera*, *chimaera*, *chimeric hybrids*, *obligate chimerism*, *homografts*, *allografts*, *xenografts*, *fusion*, *coalescence*. We specifically looked for chimerism in 12 obligately multicellular taxa for which we knew their highest levels of tumour invasiveness (vertebrata, tunicata, protostomia, placozoa, ctenophora, echinodermata, cnidaria, porifera, basidiomycota, ascomycota, embryophyta, and rhodophyta) [107,111,150] (Table 1), and in 18 terrestrial mammalian species (Table 2) for which we had data on malignancy prevalence and neoplasia prevalence.

We define chimera as an obligately multicellular organism composed of non-clonal cells not originating from mutations within the organism. Therefore by this definition, we exclude the following examples: non-genetically-examined chimeric fossil records, epigenetic phenotypic chimeras, facultative chimeras (e.g., an organism with its microbiome, including the fungome and virome), genetically modified organisms (GMOs), chimeras originating from a chimeric zygote by the fusion of two sperms with an egg (e.g. [151,152]), hybrids, epigenetic polymorphisms, conjoined twins, interspecific embryo transfers without having tested for microchimerism, multinucleated cells in fungi and red algae, plant chimeras originating from mutations within the organism during development, chimeric cells that are transmissible cancers, chimeric antibodies, no chimerism studies performed in a particular species.

### Levels of chimerism

We categorised organisms into different levels of chimerism from lowest to highest, as no chimerism (0), accepts cells from a close relative (e.g., twin or mother) (1), accepts cells from the same species other than a close relative (2), and accepts cells from different species (3). We restricted these data only to cases of obligately multicellular organisms accepting cells and maintaining them beyond reproductive age, if reported. If there was variation in the level of chimerism among literature in a taxon, we classified that taxon according to the highest level of chimerism reported for that taxon.

### Coding of chimerism levels

We recruited three biology/psychology undergraduates from Arizona State University to code the examples of highest levels of chimerism the lead author found in the existing literature into the categories described above. We gave each individual a spreadsheet with: (1) the definition of chimerism (“An obligately multicellular organism composed of non-clonal cells not originating from mutations within the organism.”); (2) a list of examples that are not chimeras (“facultative multicellular organisms, genetic hybrids (e.g., mule), epigenetic polymorphisms, conjoined twins, missing fossil evidence”); (3) the genus names/clade/division of the organisms in our database; (4) an empty column for the individuals to write the highest level of chimerism observed in each taxon; (5) a list of literature and advice to search for more examples in the literature; (6) empty columns for the individuals to complete whether experiments were performed early or later in development, and whether chimerism was natural or experimentally-induced; and (7) an empty column for the individuals to write notes or comments. The individuals completed the task within three weeks. The final highest chimerism level for each taxon that we report in this article is based on the “highest level of chimerism observed” score given by the majority of coders. For example, if among us the “highest level of chimerism observed” we gave for a taxon was 1, 1, 1, 2 (one score from each of the three undergraduate coders and the lead author), then the final “highest level of chimerism observed” for that taxon is 1. If one level did not dominate (e.g., 1, 1, 2, 2), then the lead author read additional literature, and decided what was the most correct “highest level of chimerism observed” for that taxon. Finally, while revising the manuscript, A.F. proof-read these scores based on the literature.

### Tumour invasiveness

S.E.K. collected tumour invasiveness data for 12 obligately multicellular taxa: vertebrata, tunicata, protostomia, placozoa, ctenophora, echinodermata, cnidaria, porifera, basidiomycota, ascomycota, embryophyta, and rhodophyta [107,111,150]. In order to create a scale of tumour invasiveness, S.E.K. classified tumour invasiveness from lowest to highest, as no cancer/cancer-like growth detected (0) (in studies that sought to find cancer/cancer-like growth), cancer-like growth (1), cancer (2), and transmissible cancer (3). If there was variation in the level of tumour invasiveness among species in a taxon, S.E.K. classified that taxon according to the highest level of tumour invasiveness found in any species of that taxon.

### Malignancy and neoplasia prevalence data collection

Within mammals, there was not enough variation in tumour invasiveness levels across the 18 mammalian species in our dataset in order to conduct a powerful analysis within this class, so we examined the malignancy prevalence and neoplasia prevalence of each mammalian species instead. We obtained malignancy prevalence and neoplasia prevalence data across dozens of terrestrial mammalian species. These data are from animals in zoos, aquariums, and/or private veterinary practices. Neoplasia includes benign or malignant tumours. Malignancy prevalence or neoplasia prevalence refers to the total malignant records of a species including non-neoplasia records or neoplasia records excluding non-neoplasia records, divided by the total records with denominators, respectively. Total records with denominators refer to data where we know the population size of a species from databases where non-neoplasia records are also available. We only used species for which we had ≥20 necropsies (supporting data). We excluded cancer records from wild animals. We also excluded infancy records from this database as there is usually high infant mortality across species that is not due to cancer. We excluded malignancy and neoplasia prevalence data for adult humans (https://ourworldindata.org) from our analyses because the malignancy and neoplasia data from animals under human care are collected from necropsies whereas the malignancy data in https://ourworldindata.org are not collected from necropsies, and because we do not have an estimate of benign prevalence in adult humans.

### Phylogenetic tree construction

To create a phylogenetic tree of the 12 obligately multicellular taxa across the tree of life and 11 mammalian species with more than ≥20 necropsies in our database, we used the Time Tree of Life (http://timetree.org/). At the end of the tips we placed chimeric taxa based on the majority of cells of that chimera. For example, if there was a sheep-human chimera of which the majority of cells were sheep cells and the minority human cells, we would place that species on a tip as a sheep, in order to make the phylogenetic tree.

### Statistical analyses

We performed all analyses in R version 4.0.5 [153]. To compare the association between highest tumour invasiveness, malignancy prevalence or neoplasia prevalence and highest chimerism levels, we used the R packages CAPER [154], phytools [155], geiger [156], tidyverse [157], and powerAnalysis (https://github.com/cran/powerAnalysis), and performed a phylogenetic generalized least squares (PGLS) model which takes into account the phylogenetic non-independence between taxa. In the analyses where the dependent variable was malignancy prevalence or neoplasia prevalence, we used a PGLS model weighted by 1/(square root of the number of necropsies per species) (from Revell [155]).

We first made two trees (phyl file); one including the above mentioned 12 obligately multicellular taxa across the tree of life and one with the 11 mammalian species, using the NCBI Tree creator (https://www.ncbi.nlm.nih.gov/Taxonomy/CommonTree/wwwcmt.cgi).

In the PGLS analyses of 12 obligately multicellular taxa across the tree of life, we set the variables highest tumour invasiveness levels (0, 1, 2, 3), and highest level of chimerism observed (0, 1, 2, 3), as numerical variables. When comparing malignancy prevalence, neoplasia prevalence and the highest level of chimerism observed across terrestrial mammalian species, we set malignancy prevalence and neoplasia prevalence as a dependent numerical variable and the highest level of chimerism observed as an independent categorical variable. The linear PGLS model was originally designed for continuous variables, however Graber [158] compared the statistical performance of different ordinal response models, and together with Matthews et al. [159] recommend using the PGLS model even when treating ordinal scaled variables as continuous.

## Supporting information

S1 Fig Taxa that originated on land do not have higher chimerism levels than taxa that originated in water (PGLS analysis, *P*-value > 0.05). An analysis across 12 obligately multicellular taxa on the tree of life. If a taxon includes both aquatic and terrestrial species, we have labelled that taxon according to its driest environment, i.e. terrestrial. We show each taxon with a flask, a globe, or both, according to whether it includes experimental chimeras, natural chimeras, or both, respectively. We use minimal jitter to improve visibility of individual taxa.
(TIF)

S1 Table Examples of chimerism across 12 obligately multicellular taxa. In the majority of cases in the literature, species reject foreign cells. The references are available in the reference list in the main article. The list of references in this table is not exhaustive since we do not mention here all the examples of graft rejection reported in the literature. The examples of chimerism in this table are rare examples of graft/foreign cell acceptance, if reported, in the literature.
(DOCX)

S2 Table Examples of chimerism in 18 mammalian species. In the majority of cases in the literature, species reject foreign cells. The references are available in the reference list in the main article. The list of references in the table is not exhaustive since we do not mention here all the examples of graft rejection reported in the literature. The examples of chimerism in this table are rare examples of graft/foreign cell acceptance, if reported, in the literature. EGFP: Enhanced green fluorescent protein; cGY: Centigray; Tg cells: A subset of T cells with a receptor for immunoglobulin G; HSC: Hematopoietic stem cells; DLA: Dog leucocyte antigen.
(DOCX)

S1 File (DOCX)

S1 Data The data across taxa used in this manuscript. (XLSX)
