# Supplementary figures and images for: Is chimerism associated with cancer across the tree of life?
Source: PLoS One. 2023 Jun 29;18(6):e0287901. doi: 10.1371/journal.pone.0287901 (PMC10309991; doi:10.1371/journal.pone.0287901)

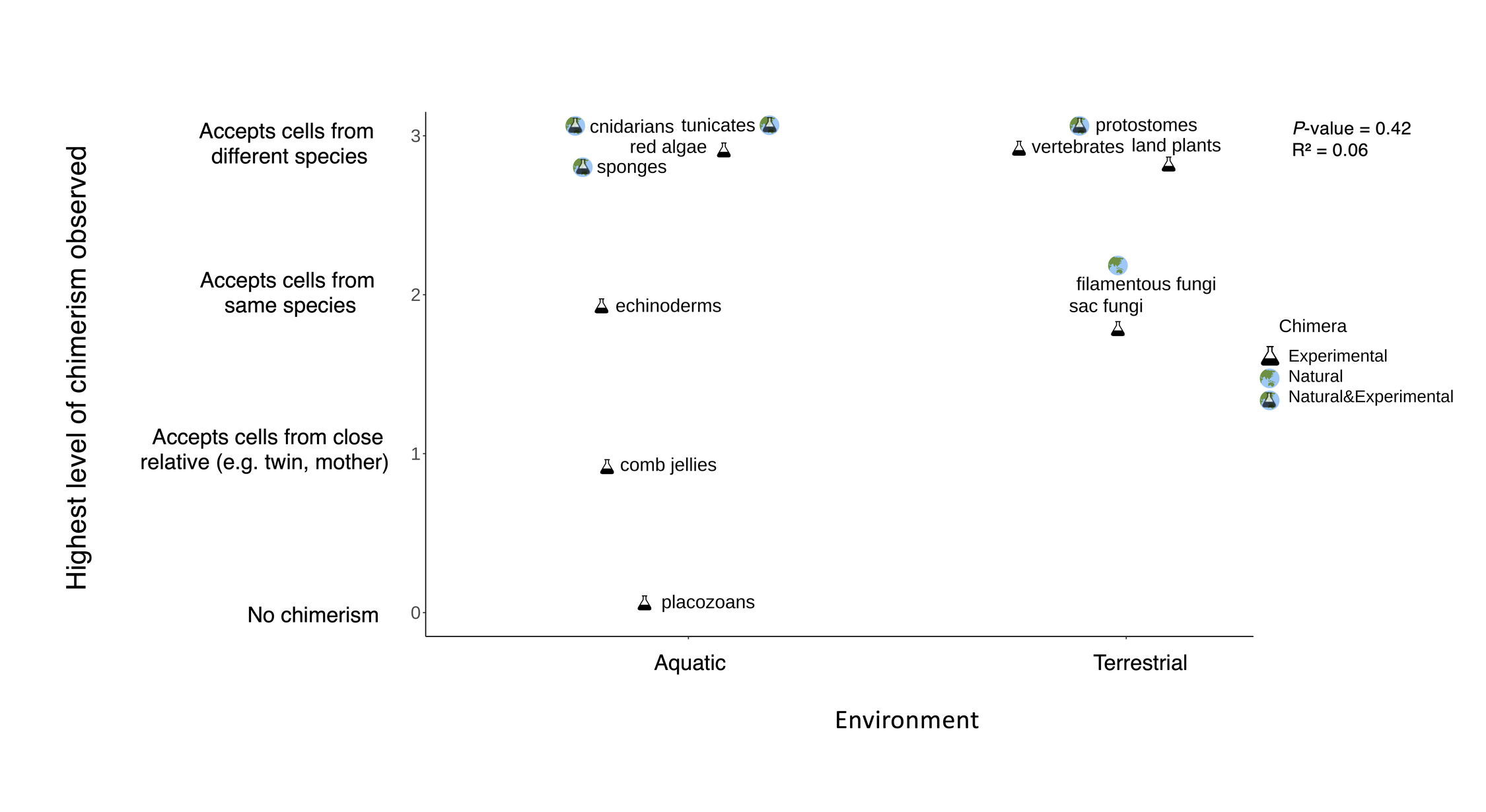

Supplement: S1 Fig — An analysis across 12 obligately multicellular taxa on the tree of life. If a taxon includes both aquatic and terrestrial species, we have labelled that taxon according to its driest environment, i.e. terrestrial. We show each taxon with a flask, a globe, or both, according to whether it includes experimental chimeras, natural chimeras, or both, respectively. We use minimal jitter to improve visibility of individual taxa. (TIF) [file pone.0287901.s001.tif]
