# Supplementary material for: Is chimerism associated with cancer across the tree of life?
Source: PLoS One. 2023 Jun 29;18(6):e0287901. doi: 10.1371/journal.pone.0287901 (PMC10309991; doi:10.1371/journal.pone.0287901)
Supplement: S1 Table — In the majority of cases in the literature, species reject foreign cells. The references are available in the reference list in the main article. The list of references in this table is not exhaustive since we do not mention here all the examples of graft rejection reported in the literature. The examples of chimerism in this table are rare examples of graft/foreign cell acceptance, if reported, in the literature. (DOCX) [file pone.0287901.s002.docx]

### **S1 Table.**

| **Taxon (common name)** | **Examples of chimerism (highest level of chimerism observed)** | **Chimerism early in development** | **Chimerism later in development** | **Natural chimerism** | **Experimental chimerism** | **Manipulation of the graft/recipient** | **For how long did the graft cells survive in the recipient?** | **How many of the graft cells survived in the recipient?** |
| --- | --- | --- | --- | --- | --- | --- | --- | --- |
| Vertebrata (vertebrates) | see Table 2 (3) | **✓**  (see Table 2) | **✓**  (see Table 2) | **✓**  (see Table 2) | **✓**  (see Table 2) | see Table 2 | see Table 2 | see Table 2 |
| Tunicata (tunicates) | chimeric colonies of 2–3 different genotypes [[67]](https://paperpile.com/c/NUwUrz/XE56z); microchimera [[79]](https://paperpile.com/c/NUwUrz/FySNW);  fusion of colonies that share at least one common allele [[75,76]](https://paperpile.com/c/NUwUrz/zMCo0+l7j5W); xenograft chimera [[77]](https://paperpile.com/c/NUwUrz/yjLrH) (3) | **✓**  “chimeras of an adult and young partner” [[78]](https://paperpile.com/c/NUwUrz/aSZ2h) | **✓**  “chimeras of an adult and young partner” [[78]](https://paperpile.com/c/NUwUrz/aSZ2h); sexually mature colonies [[76]](https://paperpile.com/c/NUwUrz/l7j5W) | **✓**  oyster grown in aquaculture facilities at the Fangar Bay [[67]](https://paperpile.com/c/NUwUrz/XE56z) | **✓**  [[67,75–77,79]](https://paperpile.com/c/NUwUrz/FySNW+zMCo0+l7j5W+XE56z+yjLrH) | experimental design “to trigger a fast fusion/non-fusion” [[67]](https://paperpile.com/c/NUwUrz/XE56z); cutting and pairing of colony fragments [[67]](https://paperpile.com/c/NUwUrz/XE56z); irradiated subclones received the grafts [[67,77]](https://paperpile.com/c/NUwUrz/yjLrH+XE56z) | 30 days [[67]](https://paperpile.com/c/NUwUrz/XE56z); 1 week [[79]](https://paperpile.com/c/NUwUrz/FySNW); months [[77]](https://paperpile.com/c/NUwUrz/yjLrH); 2 months after the fusion of colonies [[75]](https://paperpile.com/c/NUwUrz/zMCo0);  stable chimeras 8–10 months after the fusions [[76]](https://paperpile.com/c/NUwUrz/l7j5W) | presence of both genotypes in a bud [[75]](https://paperpile.com/c/NUwUrz/zMCo0); whole mass of gonads, as well as the soma, came from the  resorbed genotype [[76]](https://paperpile.com/c/NUwUrz/l7j5W); cell lineage parasitism [[160]](https://paperpile.com/c/NUwUrz/FQQv3) |
| Protostomia (protostomes) | heart allografts inside the snail *B. glabrata* [[80]](https://paperpile.com/c/NUwUrz/kK4Ul); allografts and xenografts inside *B. glabrata* [[81]](https://paperpile.com/c/NUwUrz/dExk6); xenografts [[82]](https://paperpile.com/c/NUwUrz/n3eky) (3) | N/A | **✓**  adult oysters [[82]](https://paperpile.com/c/NUwUrz/n3eky); “parents were used as donors” [[83]](https://paperpile.com/c/NUwUrz/IVMnn) | N/A | **✓**  [[80–83]](https://paperpile.com/c/NUwUrz/n3eky+kK4Ul+dExk6+IVMnn) | mesh made oysters less likely to  reject implanted nuclei [[82,161]](https://paperpile.com/c/NUwUrz/gflbV+n3eky); air-drying or incision with collagen-based skin-like scaffold to allow wound healing after transplantation [[80]](https://paperpile.com/c/NUwUrz/kK4Ul) | 6 months [[80]](https://paperpile.com/c/NUwUrz/kK4Ul); 6  months [[81]](https://paperpile.com/c/NUwUrz/dExk6); 14 days after grafting [[83]](https://paperpile.com/c/NUwUrz/IVMnn) | DNA from the donor oyster detected in the pearl sac [[162]](https://paperpile.com/c/NUwUrz/AqbXs); whether “the DNA detected is actively  transcribed, is yet to be determined.” [[82]](https://paperpile.com/c/NUwUrz/n3eky); implanted hearts continued to beat throughout the study [[80]](https://paperpile.com/c/NUwUrz/kK4Ul) |
| Placozoa (placozoans) | reaggregation of cells in *Trichoplax adhaerens*, no evidence that this new organism is functional [[84]](https://paperpile.com/c/NUwUrz/gOBrK) (0) | N/A | N/A | N/A | **✓**  [[84]](https://paperpile.com/c/NUwUrz/gOBrK) | they used dissociation medium, washed it out, suspended the cell complex and passed it through a gauze, and then they observed the free cells and small complexes reaggregate [[84]](https://paperpile.com/c/NUwUrz/gOBrK) | some reaggregates  lived for weeks and “led to apparently normal *Trichoplax* capable of further growth” [[84]](https://paperpile.com/c/NUwUrz/gOBrK) | N/A |
| Ctenophora (comb jellies) | grafting experiments [[85,86]](https://paperpile.com/c/NUwUrz/1KEXn+9xS7w): “coordination of the plates as well as the feeding reactions at the one mouth were similar to these processes of a normal animal.”, “animal consisting of the mid-pieces of four animals” [[85]](https://paperpile.com/c/NUwUrz/9xS7w) (1) | N/A | N/A | N/A | **✓**  [[85,86]](https://paperpile.com/c/NUwUrz/1KEXn+9xS7w) | “grafts were held in place by strands of cotton for about two hours to permit sufficient healing.” [[86]](https://paperpile.com/c/NUwUrz/1KEXn) | “Fusion took place within three days and the graft continued to live as a single animal.” [[85]](https://paperpile.com/c/NUwUrz/9xS7w); within two days, “Within ten days after the transplant had healed in the host, it had been completely absorbed.” [[86]](https://paperpile.com/c/NUwUrz/1KEXn) | “each piece maintained its identity by forming a mouth, by coordinating its own plate movement, and by regenerating auricles and lobes” [[85]](https://paperpile.com/c/NUwUrz/9xS7w) |
| Echinodermata (echinoderms) | viable allografts [[63]](https://paperpile.com/c/NUwUrz/o5Ilz); rejection of allografts, but autografts remained fully viable [[87]](https://paperpile.com/c/NUwUrz/hAKVn) (2) | N/A | **✓**  adult sea cucumbers and sea stars [[63]](https://paperpile.com/c/NUwUrz/o5Ilz) | N/A | **✓**  [[63,87,88]](https://paperpile.com/c/NUwUrz/o5Ilz+hAKVn+qXvmx) | animals were anesthetized before grafting, “initial fusion of the graft and subsequent healing appeared to be promoted by a light dusting of the contact zone with tetracycline wound powder and absorbable gelatin powder” [[63]](https://paperpile.com/c/NUwUrz/o5Ilz) | “allografts survived for more than 100 days but all showed slow rejection”, grafts survived from 129 to 185 days [[63]](https://paperpile.com/c/NUwUrz/o5Ilz); more than 300 days, 110 days [[87]](https://paperpile.com/c/NUwUrz/hAKVn); an average of 341.8 days [[88]](https://paperpile.com/c/NUwUrz/qXvmx) | allografts “were gradually resorbed by ingrowth of recipient tissue” [[87]](https://paperpile.com/c/NUwUrz/hAKVn) |
| Cnidaria (cnidarians) | allogeneic chimeras [[69,71]](https://paperpile.com/c/NUwUrz/nwiZR+CJg3i); three colonies had “two genotypes that each differed by two or more alleles” [[70]](https://paperpile.com/c/NUwUrz/nAtcZ); chimeras with  distinct genotypes, unrelated genotypes fused forming mature colonies [[68]](https://paperpile.com/c/NUwUrz/doUZ8) (3) | **✓**  juvenile soft coral species [[69]](https://paperpile.com/c/NUwUrz/CJg3i); “relatively young coral  colonies [[70]](https://paperpile.com/c/NUwUrz/nAtcZ); juveniles [[68]](https://paperpile.com/c/NUwUrz/doUZ8) | **✓**  juveniles and adults [[69]](https://paperpile.com/c/NUwUrz/CJg3i); adult corals [[70]](https://paperpile.com/c/NUwUrz/nAtcZ); adult and juvenile colonies [[68]](https://paperpile.com/c/NUwUrz/doUZ8) | **✓**  from Canada [[71]](https://paperpile.com/c/NUwUrz/nwiZR); natural colonies from the northern Gulf of Eilat [[69]](https://paperpile.com/c/NUwUrz/CJg3i); colonies  from Magnetic Island [[70]](https://paperpile.com/c/NUwUrz/nAtcZ); natural populations [[68]](https://paperpile.com/c/NUwUrz/doUZ8) | **✓**  [[89]](https://paperpile.com/c/NUwUrz/6uH5R) | “to increase the likelihood of detecting genetic  variability at the colony level, branches were sampled as far away from each other as possible across the colony.” [[68,70]](https://paperpile.com/c/NUwUrz/nAtcZ+doUZ8) | up to 450 days [[69]](https://paperpile.com/c/NUwUrz/CJg3i); “more than 4 months after chimeras were made.” [[89]](https://paperpile.com/c/NUwUrz/6uH5R) | “partners shared layers of endoderm, mesoglea and ectoderm, and the gastrovascular cavity” [[71]](https://paperpile.com/c/NUwUrz/nwiZR); “movement of cells from one partner to the other” [[69]](https://paperpile.com/c/NUwUrz/CJg3i); “chimeras had *H. attenuata* epithelial cells and *P. oligactis* interstitial cell lineage” [[89]](https://paperpile.com/c/NUwUrz/6uH5R) |
| Porifera (sponges) | not all individuals fused [[73,163]](https://paperpile.com/c/NUwUrz/n9CPT+5ifzi); “fuse in twos or threes or in larger number up to and over one hundred.” [[91]](https://paperpile.com/c/NUwUrz/fWju5) ; bispecific conglomerates [[72,90]](https://paperpile.com/c/NUwUrz/TUYVi+pCGpq) (3) | **✓**  larvae [[73]](https://paperpile.com/c/NUwUrz/5ifzi); fusion between larvae [[91]](https://paperpile.com/c/NUwUrz/fWju5); sibling larvae [[92]](https://paperpile.com/c/NUwUrz/ctw8R) | **✓**  “Both, adult and larval DNA was extracted” [[73]](https://paperpile.com/c/NUwUrz/5ifzi) | **✓**  naturally occurring bispecific chimeras [[72]](https://paperpile.com/c/NUwUrz/TUYVi); natural populations of marine sponges [[73]](https://paperpile.com/c/NUwUrz/5ifzi) | **✓**  [[90–93]](https://paperpile.com/c/NUwUrz/pCGpq+JpCsQ+fWju5+ctw8R) | fusion at a critical time when “the ciliated epithelium is being replaced by the permanent flat epithelium”, “with pipette and needle coaxed together into a clump.” [[91]](https://paperpile.com/c/NUwUrz/fWju5); “pairs of sibling larvae were forced to settle in contact, they fused in all cases.” [[92]](https://paperpile.com/c/NUwUrz/ctw8R) | 48 hours after the beginning of the culture, 72 hours [[93]](https://paperpile.com/c/NUwUrz/JpCsQ); 50 days [[92]](https://paperpile.com/c/NUwUrz/ctw8R) | “the Oxymycale portion comprises the bulk of the specimen, the Sigmadocia part being about l0 per cent of the whole” [[72]](https://paperpile.com/c/NUwUrz/TUYVi); “small aggregates were formed and sometimes patches of cells of the two species loosely adhered.” [[90]](https://paperpile.com/c/NUwUrz/pCGpq) |
| Basidiomycota (filamentous fungi) | “Ten cells isolated from a single basidiome produced nine different genotypes when analyzed for variation at six nuclear loci.” [[74]](https://paperpile.com/c/NUwUrz/1r6C7) (2) | N/A | N/A | **✓**  samples collected from a lawn in Massachusett [[74]](https://paperpile.com/c/NUwUrz/1r6C7) | N/A | N/A | N/A | N/A |
| Ascomycota (sac fungi) | “branching and fusion within the *N. crassa* hyphal network mix genetically diverse nuclei and create well-mixed conidial spores” [[95]](https://paperpile.com/c/NUwUrz/if9ST); hyphae “fuse within a colony, but also between colonies of the same species.” [[94]](https://paperpile.com/c/NUwUrz/ikvDa) (2) | **✓**  fusion of germinating conidia with nearby mature hyphae [[95]](https://paperpile.com/c/NUwUrz/if9ST) | **✓**  fusion of germinating conidia with nearby mature hyphae [[95]](https://paperpile.com/c/NUwUrz/if9ST) | N/A | **✓**  [[94,95]](https://paperpile.com/c/NUwUrz/if9ST+ikvDa) | “conidia were used to initiate heterokaryotic mycelia.” [[95]](https://paperpile.com/c/NUwUrz/if9ST) | N/A | N/A |
| Embryophyta (land plants) | “four of the 1321 plants regenerated from chimeral callus were chimeras” [[100]](https://paperpile.com/c/NUwUrz/h2lOp); intraspecific chimeras [[100,164]](https://paperpile.com/c/NUwUrz/k2Hlu+h2lOp); interspecific chimeras [[96–98]](https://paperpile.com/c/NUwUrz/2KpHF+ZeLSy+r9TiH) (3) | **✓**  [[99]](https://paperpile.com/c/NUwUrz/NMhWU); Ten-day-old stenle seedlings [[100]](https://paperpile.com/c/NUwUrz/h2lOp); young plants of two species [[98]](https://paperpile.com/c/NUwUrz/r9TiH) | N/A | N/A | **✓**  [[98,100]](https://paperpile.com/c/NUwUrz/r9TiH+h2lOp) | they used the semidominant aurea mutant of tobacco, interspecific cultures in vitro were placed in media  favouring only *N. tabacum* shoot formation, “Graft unions were secured with budding rubbers” [[100]](https://paperpile.com/c/NUwUrz/h2lOp) | 46, 53,  70 and 73 days after decapitation [[97]](https://paperpile.com/c/NUwUrz/ZeLSy) | “Eventually the *N. glauca* tissue in the original menstem was replaced by *N tabacum*” [[97]](https://paperpile.com/c/NUwUrz/ZeLSy); “The two lower leaves are partly composed of one layer of *luteum*  over tomato and partly of pure *luteum*” [[98]](https://paperpile.com/c/NUwUrz/r9TiH) |
| Rhodophyta (red algae) | “interindividual fusions in red and brown algal species” [[101]](https://paperpile.com/c/NUwUrz/juDYs) (3) | **✓**  [[101]](https://paperpile.com/c/NUwUrz/juDYs) | N/A | N/A | **✓**  laboratory-built bicolor chimeras [[101]](https://paperpile.com/c/NUwUrz/juDYs) | N/A | 30–45 days [[101]](https://paperpile.com/c/NUwUrz/juDYs) | “the cell mix (bicolor with green and red) appeared to extend from the base up to 25% of the basalmost portion of the shorter axes”, “the mixed tissue reached 10%–15% the axes length”, “a combination of red and green alleles (chimeric tissues) was found at the apical portions of the chimeric axis” [[101]](https://paperpile.com/c/NUwUrz/juDYs) |

###

## **Supporting references** (not found in the main text reference list)

160. [Rinkevich B. Natural chimerism in colonial urochordates. J Exp Mar Bio Ecol. 2005;322: 93–109.](http://paperpile.com/b/NUwUrz/FQQv3)

161. [Gervis MH, Sims NA. The Biology and Culture of Pearl Oysters (Bivalvia Pteriidae). WorldFish; 1992.](http://paperpile.com/b/NUwUrz/gflbV)

162. [Arnaud-Haond S, Goyard E, Vonau V, Herbaut C, Prou J, Saulnier D. Pearl formation: persistence of the graft during the entire process of biomineralization. Mar Biotechnol . 2007;9: 113–116.](http://paperpile.com/b/NUwUrz/AqbXs)

163. [Aanen DK, Debets AJM, de Visser JAGM, Hoekstra RF. The social evolution of somatic fusion. Bioessays. 2008;30: 1193–1203.](http://paperpile.com/b/NUwUrz/n9CPT)

164. [Carlson PS. Mitotic crossing-over in a higher plant. Genet Res . 1974. Available:](http://paperpile.com/b/NUwUrz/k2Hlu) <https://www.cambridge.org/core/journals/genetics-research/article/mitotic-crossingover-in-a-higher-plant/3CAD4DD6E15037CB2EDB46895CDF7ADE>
