# Supplementary material for: Is chimerism associated with cancer across the tree of life?
Source: PLoS One. 2023 Jun 29;18(6):e0287901. doi: 10.1371/journal.pone.0287901 (PMC10309991; doi:10.1371/journal.pone.0287901)
Supplement: S2 Table — In the majority of cases in the literature, species reject foreign cells. The references are available in the reference list in the main article. The list of references in the table is not exhaustive since we do not mention here all the examples of graft rejection reported in the literature. The examples of chimerism in this table are rare examples of graft/foreign cell acceptance, if reported, in the literature. EGFP: Enhanced green fluorescent protein; cGY: Centigray; Tg cells: A subset of T cells with a receptor for immunoglobulin G; HSC: Hematopoietic stem cells; DLA: Dog leucocyte antigen. (DOCX) [file pone.0287901.s003.docx]

### **S2 Table.**

| **Mammalian species (common name)** | **Examples of chimerism (highest level of chimerism observed)** | **Chimerism early in development** | **Chimerism later in development** | **Natural chimerism** | **Experimental chimerism** | **Manipulation of the graft/recipient** | **For how long did the graft cells survive in the recipient?** | **How many of the graft cells survived in the recipient?** |
| --- | --- | --- | --- | --- | --- | --- | --- | --- |
| *Saguinus oedipus* (cotton-top tamarin) | microchimerism [[102]](https://paperpile.com/c/NUwUrz/xVpC7) (1) | **✓**  [[102]](https://paperpile.com/c/NUwUrz/xVpC7) | N/A | **✓**  “naturally occurring chimeric bone marrow” [[102]](https://paperpile.com/c/NUwUrz/xVpC7) | N/A | N/A | N/A | “both the lymphocyte and monocyte/macrophage populations of these animals are chimeric” [[102]](https://paperpile.com/c/NUwUrz/xVpC7) |
| *Rattus norvegicus* (common rat) | allogeneic chimeras [[110]](https://paperpile.com/c/NUwUrz/8pJGe); retinae from mice transplanted to the midbrain of rats [[112]](https://paperpile.com/c/NUwUrz/ceDKb); retinae transplanted into the brains of rats [[111]](https://paperpile.com/c/NUwUrz/mvuPe) (3) | **✓**  embryonic mice as donors and neonatal rats as hosts [[112]](https://paperpile.com/c/NUwUrz/ceDKb); fetal retinae from rats [[111]](https://paperpile.com/c/NUwUrz/mvuPe) | N/A | N/A | **✓**  [[110–112]](https://paperpile.com/c/NUwUrz/ceDKb+mvuPe+8pJGe) | bone marrow cells conditioned with 1100 cGy, the bone marrow was T cell depleted [[110]](https://paperpile.com/c/NUwUrz/8pJGe); “recipients were unilaterally enucleated at this time in order to enhance innervation of the host by the transplant” [[112]](https://paperpile.com/c/NUwUrz/ceDKb) | 300 days after transplantation, ≥14 months [[110]](https://paperpile.com/c/NUwUrz/8pJGe) | grafts in “areas of the brain stem normally innervated by the eye” [[112]](https://paperpile.com/c/NUwUrz/ceDKb); “well-formed grafts containing numerous rosettes and ganglion cells”, “The transplanted retinae survived, differentiated, and grew to  2 mm or more in diameter.” [[111]](https://paperpile.com/c/NUwUrz/mvuPe) |
| *Mus musculus* (house mouse) | “Tetraparental mice are formed by the in vitro fusion of two eight-  cell stage mouse embryos” [[114]](https://paperpile.com/c/NUwUrz/mdvUI); human cells implanted in the brain of  mice, “immunotolerance of the embryonic brain.” [[113]](https://paperpile.com/c/NUwUrz/jBFol) (3) | **✓**  [[113–115]](https://paperpile.com/c/NUwUrz/jBFol+mdvUI+MFgFg) | N/A | N/A | **✓**  [[113–115]](https://paperpile.com/c/NUwUrz/jBFol+mdvUI+MFgFg) | “(human embryonic stem cells) were cocultured with mouse embryonic fibroblasts in a defined medium and were immunoreactive for (specific) undifferentiated markers” [[113]](https://paperpile.com/c/NUwUrz/jBFol) | “Two months after transplantation, 18 months [[113]](https://paperpile.com/c/NUwUrz/jBFol); 2 weeks following engraftment, “by 3–5 weeks had appropriately differentiated into oligodendrocytes and astrocytes” [[115]](https://paperpile.com/c/NUwUrz/MFgFg) | “Transplanted cells were identified in brain slices by EGFP fluorescence” , “an average of six EGFP cells were found in each 40-m brain microtome section.”, “0.1% of the brain cells are of human origin.” [[113]](https://paperpile.com/c/NUwUrz/jBFol) |
| *Sarcophilus harrisii* (Tasmanian devil) | “All successful allografts were acutely rejected” [[116]](https://paperpile.com/c/NUwUrz/s1qS2) (0) | N/A | **✓**  three year old females, three year old male [[116]](https://paperpile.com/c/NUwUrz/s1qS2) | N/A | **✓**  [[116]](https://paperpile.com/c/NUwUrz/s1qS2) | “Surgical glue was placed on the borders of the grafts to secure the  skin.”, they administered pain relief medication  “after skin graft surgery” [[116]](https://paperpile.com/c/NUwUrz/s1qS2) | “14 days after surgery”,  “progressed to very severe rejection on Day 21.” [[116]](https://paperpile.com/c/NUwUrz/s1qS2) | “necrosis associated with polymorphonuclear cell infiltration, surface parakeratosis and fibrin deposition.” [[116]](https://paperpile.com/c/NUwUrz/s1qS2) |
| *Ovis aries* (sheep) | embryonically derived human hematopoietic stem cells in sheep [[117,118]](https://paperpile.com/c/NUwUrz/WTxjW+Dbctf) (3) | **✓**  “Using the fetal transplant method, he injects embryonically derived human hematopoietic stem cells into sheep.” [[117,118]](https://paperpile.com/c/NUwUrz/WTxjW+Dbctf) | N/A | N/A | **✓**  [[117,118]](https://paperpile.com/c/NUwUrz/WTxjW+Dbctf) | “the  HSC are transplanted early in gestation when the recipient is  still largely immunologically naive.” [[118]](https://paperpile.com/c/NUwUrz/Dbctf) | seven years later  [[117,118]](https://paperpile.com/c/NUwUrz/WTxjW+Dbctf); “the sheep maintained their  chimeric status for at least 9 months.” [[118]](https://paperpile.com/c/NUwUrz/Dbctf) | “an animal with organs, including the liver, heart and pancreas, that are 15% human.” [[117,118]](https://paperpile.com/c/NUwUrz/WTxjW+Dbctf) |
| *Bos taurus* (cattle) | microchimerism [[103]](https://paperpile.com/c/NUwUrz/ZebUW); Y chro-  mosome-specific DNA in naturally mated heifers “carrying conventional bull calves” and a transgene-specific sequence in “cows  carrying transgenic fetuses.” [[119]](https://paperpile.com/c/NUwUrz/5I47D) (1) | **✓**  vascular anastomose [[103]](https://paperpile.com/c/NUwUrz/ZebUW) ; fetuses [[119]](https://paperpile.com/c/NUwUrz/5I47D) | **✓**  [[103]](https://paperpile.com/c/NUwUrz/ZebUW) | **✓**  [[103]](https://paperpile.com/c/NUwUrz/ZebUW);  “naturally mated heifers” [[119]](https://paperpile.com/c/NUwUrz/5I47D) | **✓**  [[119]](https://paperpile.com/c/NUwUrz/5I47D) | “conventional recipient cows pregnant after non-surgical transfer of a single Tg embryo produced by  in vitro fertilization of transvaginally recovered oocytes” [[119,120]](https://paperpile.com/c/NUwUrz/2He3j+5I47D) | “many of the twins in this study were adults when they were tested” [[103]](https://paperpile.com/c/NUwUrz/ZebUW); “fetal DNA found in the maternal circulation up to 4 months postpartum” [[119]](https://paperpile.com/c/NUwUrz/5I47D) | “the magnitude of the microchimerism is in the order of six circulating male cells or their corresponding DNA contents (if cell-free) per mL of maternal blood and about 36 Tg cells, respectively.” [[119]](https://paperpile.com/c/NUwUrz/5I47D) |
| *Sus scrofa* (wild boar) | human cells in pigs (3) | **✓**  “hematopoietic stem cells were engrafted in pigs.” [[121]](https://paperpile.com/c/NUwUrz/hsK6E) | N/A | N/A | **✓**  [[121]](https://paperpile.com/c/NUwUrz/hsK6E) | “injecting fetal pigs with 5 × 10^7 human T cell-depleted bone marrow cells” [[121]](https://paperpile.com/c/NUwUrz/hsK6E) | “long-term engraftment (>1 year) of human  cells in pigs.” [[121]](https://paperpile.com/c/NUwUrz/hsK6E) | N/A |
| *Acinonyx jubatus* (cheetah) | accepts allograft, “14 reciprocal skin grafts between unrelated cheetahs were accepted”, skin grafts from domestic cats were rapidly rejected [[122]](https://paperpile.com/c/NUwUrz/5YmuK) (2) | N/A | **✓**  [[122]](https://paperpile.com/c/NUwUrz/5YmuK) | N/A | **✓**  [[122]](https://paperpile.com/c/NUwUrz/5YmuK) | “antibiotics were administered and the surgical area was bandaged” [[122]](https://paperpile.com/c/NUwUrz/5YmuK) | “allograft and autograft were virtually indistinguishable 2 weeks after surgery”, “studies were terminated early (day 23)” [[122]](https://paperpile.com/c/NUwUrz/5YmuK) | N/A |
| *Canis lupus familiaris* (domestic dog) | “Bone marrow allografts performed in pairs of dogs bearing identical DLA haplotypes” [[123]](https://paperpile.com/c/NUwUrz/dKZAl); fetal microchimerism [[104]](https://paperpile.com/c/NUwUrz/NCrtQ); skin allograft tolerance [[124]](https://paperpile.com/c/NUwUrz/lGjTl) (2) | **✓**  [[104]](https://paperpile.com/c/NUwUrz/NCrtQ) | **✓**  young adult dogs [[124]](https://paperpile.com/c/NUwUrz/lGjTl); adult beagles [[123]](https://paperpile.com/c/NUwUrz/dKZAl) | **✓**  [[104]](https://paperpile.com/c/NUwUrz/NCrtQ) | **✓**  [[123,124]](https://paperpile.com/c/NUwUrz/lGjTl+dKZAl) | “nonmyeloablative conditioning (200 cGy TBI) and transient immunosuppression” [[124]](https://paperpile.com/c/NUwUrz/lGjTl); “Radiation chimeras given bone marrow” [[123]](https://paperpile.com/c/NUwUrz/dKZAl) | “4 weeks posttransplantation”, for at least 76 weeks, “There was long-term (>5 yrs) renal allograft survival” [[124]](https://paperpile.com/c/NUwUrz/lGjTl); 10-25 days [[123]](https://paperpile.com/c/NUwUrz/dKZAl) | “T and B cells contained donor-type cells” [[124]](https://paperpile.com/c/NUwUrz/lGjTl); “The actual rate of microchimerism may be higher, as the assay performed here would not detect female  Microchimerism.” [[123]](https://paperpile.com/c/NUwUrz/dKZAl) |
| *Papio hamadryas* (Hamadryas baboon) | xenotransplantation, “No hyperacute rejection de-  veloped” [[125]](https://paperpile.com/c/NUwUrz/fVlKA); “Seven pig-to-baboon orthotopic liver xeno-  grafts are reported.” [[126]](https://paperpile.com/c/NUwUrz/CFDmO) (3) | **✓**  “piglets were used as donors to recipient baboons” [[126]](https://paperpile.com/c/NUwUrz/CFDmO) | N/A | N/A | **✓**  [[125,126]](https://paperpile.com/c/NUwUrz/CFDmO+fVlKA) | “treated with a chronic immunosuppressive regimen.”, “Thymic irradiation (700 cGy)”, “Three baboons were treated with aspirin” [[125]](https://paperpile.com/c/NUwUrz/fVlKA); “The remaining three animals, which were given human fibrinogen, did not bleed.” [[126]](https://paperpile.com/c/NUwUrz/CFDmO) | “one graft survived up to 6 months after transplantation.”, 78 and 179 days [[125]](https://paperpile.com/c/NUwUrz/fVlKA); survival of xenografts up to 3 ½ days [[126]](https://paperpile.com/c/NUwUrz/CFDmO) | N/A |
| *Homo sapiens* (human) | allogeneic organ transplantation [[127]](https://paperpile.com/c/NUwUrz/tqopO); blood group chimerism in twins and triplets [[105]](https://paperpile.com/c/NUwUrz/5aLcI); xenograft from baboon to human [[128]](https://paperpile.com/c/NUwUrz/3Odfo) (3) | **✓**  microchimerism [[23,24,105,106]](https://paperpile.com/c/NUwUrz/hPEAE+5aLcI+Z5Qbx+TTJcC) | **✓**  recipient was a 35-year old human, the donor was a “15-year-old male baboon” [[128]](https://paperpile.com/c/NUwUrz/3Odfo); microchimerism between mother and fetus during pregnancy [[23,24,106]](https://paperpile.com/c/NUwUrz/hPEAE+Z5Qbx+TTJcC) | **✓**  natural microchimerism [[23,24,105,106,127]](https://paperpile.com/c/NUwUrz/hPEAE+tqopO+5aLcI+Z5Qbx+TTJcC) | **✓**  [[127,128]](https://paperpile.com/c/NUwUrz/tqopO+3Odfo) | immunosuppression “to mitigate preformed antigraft antibody syndromes and  cellular rejection”, “20 units of blood  were given during the 11 h operation.” [[128]](https://paperpile.com/c/NUwUrz/3Odfo) | 70 days [[128]](https://paperpile.com/c/NUwUrz/3Odfo); “fetal cells have been found to persist for years, probably for a lifetime, in the circulation of healthy women” [[106]](https://paperpile.com/c/NUwUrz/hPEAE); “Twins share cells in the womb and can harbor these cells into adulthood” [[23]](https://paperpile.com/c/NUwUrz/Z5Qbx) | fetal cells in the blood of mothers with scleroderma disease: “an average of seven male cells per 10 milliliters of blood.”, “the level of fetal cells is about one in 1 million cells in maternal circulation,”, “a 48-year-old  mother who had a goiter removed. To her  surprise upon examining the removed goiter  tissue, Bianchi discovered that one whole section of the woman’s thyroid was predominantly male, presumably from her son.” [[23]](https://paperpile.com/c/NUwUrz/Z5Qbx) |
| *Callithrix jacchus* (common marmoset) | blood chimerism between twins and triplets [[107]](https://paperpile.com/c/NUwUrz/QfHqo) (1) | **✓**  tissues from “animals that had died at birth or had to be euthanized for reasons of bad health.” [[107]](https://paperpile.com/c/NUwUrz/QfHqo) | N/A | **✓**  “from a captive colony kept at the Psychological Institute,  University of Zurich” [[107]](https://paperpile.com/c/NUwUrz/QfHqo) | N/A | N/A | age at birth or age when euthanized [[107]](https://paperpile.com/c/NUwUrz/QfHqo) | N/A |
| *Didelphis virginiana* (Virginia opossum) | maternal allograft, “none of the 24 young less than 12 days of age rejected the maternal allografts” [[129]](https://paperpile.com/c/NUwUrz/yNNLp) (1) | **✓**  grafts “were taken from the maternal ear skin and placed on the dorsum of the pouch young at ages varying from 3-17 days” [[129]](https://paperpile.com/c/NUwUrz/yNNLp) | **✓**  grafts from maternal skin [[129]](https://paperpile.com/c/NUwUrz/yNNLp) | N/A | **✓**  [[129]](https://paperpile.com/c/NUwUrz/yNNLp) | “the mother (was) anesthetised with sodium pentobarbital” [[129]](https://paperpile.com/c/NUwUrz/yNNLp) | “at least 80 days in most cases” [[129]](https://paperpile.com/c/NUwUrz/yNNLp) | N/A |
| *Oryctolagus cuniculus* (European rabbit) | xenografted rat tissue [[130]](https://paperpile.com/c/NUwUrz/GvAtL) (3) | N/A | N/A | N/A | **✓**  [[130]](https://paperpile.com/c/NUwUrz/GvAtL) | N/A | “rat hippocampal grafts developing for 8 weeks in the rabbit septum” [[130]](https://paperpile.com/c/NUwUrz/GvAtL) | “grafts significantly increased in their volume (600 to 800% of the initial value). Typical pyramidal neurons were present in the grafted hippocampus, though their organization into a typical layer was absent” [[130]](https://paperpile.com/c/NUwUrz/GvAtL) |
| *Equus ferus* (wild horse) | microchimerism, “ blood chimerism occurred in 4 out of 5 cases with identical blood group” [[108]](https://paperpile.com/c/NUwUrz/RfS0E) (1) | **✓**  [[108]](https://paperpile.com/c/NUwUrz/RfS0E) | N/A | **✓**  [[108]](https://paperpile.com/c/NUwUrz/RfS0E) | N/A | N/A | "fusion of the chorial sacs was found to have developed in two  cases of mummified foetus which were at about the 6th month of gestation.” [[108]](https://paperpile.com/c/NUwUrz/RfS0E) | N/A |
| *Dasypus novemcinctus* (armadillo) | skin grafts accepted between monozygotic littermates [[131]](https://paperpile.com/c/NUwUrz/5hdG3) (1) | **✓**  “each animal both donated a graft to and received a graft from each of its littermates” [[131]](https://paperpile.com/c/NUwUrz/5hdG3) | N/A | N/A | **✓**  [[131]](https://paperpile.com/c/NUwUrz/5hdG3) | “grafts were redressed at the early inspections to protect them from trauma.” [[131]](https://paperpile.com/c/NUwUrz/5hdG3) | 20 days, 50 days after grafting, “on the 85th postoperative day, when the observation period was terminated.” [[131]](https://paperpile.com/c/NUwUrz/5hdG3) | N/A |
| *Mesocricetus auratus* (Syrian hamster) | skin homografts between individuals of completely unrelated stocks [[132]](https://paperpile.com/c/NUwUrz/vlXUS) (2) | N/A | **✓**  exchanging grafts between adult animal [[132]](https://paperpile.com/c/NUwUrz/vlXUS) | N/A | **✓**  [[132]](https://paperpile.com/c/NUwUrz/vlXUS) | “each animal received a single graft fitted into an appropriately sized bed on the lateral thoracic wall.” [[132]](https://paperpile.com/c/NUwUrz/vlXUS) | 200 days, 24 to 140 days [[132]](https://paperpile.com/c/NUwUrz/vlXUS) | N/A |
| *Macaca mulatta* (rhesus macaque) | maternal microchimerism [[109]](https://paperpile.com/c/NUwUrz/n1aTA) (1) | **✓**  fetal blood from the early third trimester [[109]](https://paperpile.com/c/NUwUrz/n1aTA) | **✓**  “maternal  blood samples were collected during gestation and at pregnancy  termination.” [[109]](https://paperpile.com/c/NUwUrz/n1aTA) | **✓**  [[109]](https://paperpile.com/c/NUwUrz/n1aTA) | N/A | N/A | 1–1.5 years of age [[109]](https://paperpile.com/c/NUwUrz/n1aTA) | “maternal microchimerism in at least one compartment (thymus, liver, spleen, lymph nodes,  and bone marrow) (range: 0.001–1.9%  chimeric cells).” [[109]](https://paperpile.com/c/NUwUrz/n1aTA) |
