# Supplementary material for: Is chimerism associated with cancer across the tree of life?
Source: PLoS One. 2023 Jun 29;18(6):e0287901. doi: 10.1371/journal.pone.0287901 (PMC10309991; doi:10.1371/journal.pone.0287901)
Supplement: S1 File — (DOCX) [file pone.0287901.s004.docx]

## **Supporting methods**

### We obtained information about the environmental origins of 12 obligately multicellular taxa, aquatic (0) or terrestrial (1), from Fisher et al. [[9]](https://paperpile.com/c/NUwUrz/X4f6a),<https://animaldiversity.org/>, and<https://www.iucnredlist.org/>. If species in a taxon lived in both aquatic and terrestrial environments, we classified that taxon according to the driest environment of any species in that taxon, i.e. terrestrial environment. In the PGLS analyses of 12 obligately multicellular taxa across the tree of life, we set the variables and environment (0, 1) as numerical variables.

## **Supporting results**

Obligately multicellular taxa that originated on land do not have higher observed chimerism levels than obligately multicellular taxa that originated in aquatic environments (S1 Fig; PGLS analysis: F-statistic = 0.67 on 1 and 10 DF, ML lambda = 1, R² = 0.06, *P*-value = 0.42).
