# Supplementary material for: Is chimerism associated with cancer across the tree of life?
Source: PLoS One. 2023 Jun 29;18(6):e0287901. doi: 10.1371/journal.pone.0287901 (PMC10309991; doi:10.1371/journal.pone.0287901)
Supplement: Attachment — Submitted filename: Response to Reviewers.docx [file pone.0287901.s006.docx]

- **1. Please ensure that your manuscript meets PLOS ONE's style requirements, including those for file naming. The PLOS ONE style templates can be found at** [**https://journals.plos.org/plosone/s/file?id=wjVg/PLOSOne_formatting_sample_main_body.pdf**](https://urldefense.com/v3/__https://journals.plos.org/plosone/s/file?id=wjVg*PLOSOne_formatting_sample_main_body.pdf__;Lw!!IKRxdwAv5BmarQ!ZTs_FvAsDDHKV6G2zrHH248tQDUnYGwr-H98JO51lxqoruI20Vk-FF4GxcKG0ayHOaaOpvTCOuPjBfI$) **and** [**https://journals.plos.org/plosone/s/file?id=ba62/PLOSOne_formatting_sample_title_authors_affiliations.pdf**](https://urldefense.com/v3/__https://journals.plos.org/plosone/s/file?id=ba62*PLOSOne_formatting_sample_title_authors_affiliations.pdf__;Lw!!IKRxdwAv5BmarQ!ZTs_FvAsDDHKV6G2zrHH248tQDUnYGwr-H98JO51lxqoruI20Vk-FF4GxcKG0ayHOaaOpvTCkHkMVR4$)
  - We updated the format of the manuscript, made sure the figures were in order, and reuploaded the figures and supplementary figure as .tiff files, according to the PLOS ONE style templates.
- **2. Thank you for stating the following financial disclosure:**

**"This work was supported in part by NIH grants U54 CA217376, U2C CA233254, P01 CA91955, and R01 CA140657 as well as CDMRP Breast Cancer Research Program Award BC132057 and the Arizona Biomedical Research Commission grant ADHS18-198847. The findings, opinions and recommendations expressed here are those of the authors and not necessarily those of the universities where the research was performed or the National Institutes of Health."**

**Please state what role the funders took in the study. If the funders had no role, please state: "The funders had no role in study design, data collection and analysis, decision to publish, or preparation of the manuscript."**

**If this statement is not correct you must amend it as needed.**

**Please include this amended Role of Funder statement in your cover letter; we will change the online submission form on your behalf.**

- We added this "The funders had no role in study design, data collection and analysis, decision to publish, or preparation of the manuscript." in the financial disclosure section of the manuscript.
- We’ve also added this "The funders had no role in study design, data collection and analysis, decision to publish, or preparation of the manuscript." in the cover letter.
- **3. Please note that supplementary tables (should remain/ be uploaded) as separate "supporting information" files.**

**[Note: HTML markup is below. Please do not edit.]**

- We have now uploaded the supplementary tables as supporting information files.
- **1. Is the manuscript technically sound, and do the data support the conclusions?**

**The manuscript must describe a technically sound piece of scientific research with data that supports the conclusions. Experiments must have been conducted rigorously, with appropriate controls, replication, and sample sizes. The conclusions must be drawn appropriately based on the data presented.**

**Reviewer #1: No**

**Reviewer #2: Partly**

- We address the reviewers’ comments as they described them in detail below.
- **2. Has the statistical analysis been performed appropriately and rigorously?**

**Reviewer #1: No**

**Reviewer #2: I Don't Know**

- We describe the statistical analyses conducted in the “Statistical Analyses” section in our Methods section. We address the more specific concerns of the reviewers about this section below.
- **3. Have the authors made all data underlying the findings in their manuscript fully available?**

**The** [**PLOS Data policy**](https://urldefense.com/v3/__http://www.plosone.org/static/policies.action*sharing__;Iw!!IKRxdwAv5BmarQ!ZTs_FvAsDDHKV6G2zrHH248tQDUnYGwr-H98JO51lxqoruI20Vk-FF4GxcKG0ayHOaaOpvTCDY07PJk$) **requires authors to make all data underlying the findings described in their manuscript fully available without restriction, with rare exception (please refer to the Data Availability Statement in the manuscript PDF file). The data should be provided as part of the manuscript or its supporting information, or deposited to a public repository. For example, in addition to summary statistics, the data points behind means, medians and variance measures should be available. If there are restrictions on publicly sharing data—e.g. participant privacy or use of data from a third party—those must be specified.**

**Reviewer #1: No**

**Reviewer #2: Yes**

- The data of this manuscript are provided in the Supporting Data.
- **Reviewer 1’s comments:**

**“However the data and approach presented in the manuscript is weak. The authors themselves state that this analysis is an ‘imprecise view of the relationship between chimerism and cancer’ which I tend to agree with.”**

- We mention this in the text because we see that there is definitely room for future work in this field. “Thus, our analysis is just a first, imprecise view of the relationship between chimerism and cancer. Future studies would benefit from testing…”. This does not weaken our study. We make an important first step in the field of associations between neoplasia prevalence/tumor invasiveness and chimerism across taxa.
- **“I question the appropriateness of the method by which the highest chimerism level was scored for taxa. No weighting is given to the amount or degree of literature support for a score, or no threshold used, within a certain taxon which might be one approach to tackle unavoidable sampling or reporting bias in a systematic literature review of this kind.”**
  - We report the literature related to each score in Tables S1 and S2. The number of references is evident in each row. We did not weigh the scoring by the amount or degree of literature support for a score because the exact chimerism-related information reported in each study can vary quite a lot between studies. That is why we used five different people to assess the scores.
- **“No attempt appears to be made to mitigate this bias although it is acknowledged. The authors do not present decision criteria used to determine whether or not a given study should be included beyond presence or absence of key words.”**
  - Five different people assessed the literature and the chimerism scores. Additionally, we provide support for the choice of each score using chimerism-supporting phrases from the literature (Tables S1 and S2).
- **“In one of the examples given, if the “highest level of chimerism observed” for a taxon was scored as a 1 by all undergraduate coders and as a ‘2’ by the lead author and expert, then the highest score remained as 1. Essentially meaning that where an expert disagreed with inexpert (by virtue of exposure/experience) undergraduates working over just a 3 week period, the undergraduate score was retained. Considering the complexity of establishing a highest degree of chimerism for an entire taxon based on synthesis of the literature, I’m not at all convinced that a scenario like this wouldn’t reflect some misunderstanding on the part of the recruited undergraduates. In particular where it is clear that some of these coders do not have undergraduate training in biology (text states some of the peer-reviewers are psychology majors). There is no validation of repeatability for this scoring system and the associations being tested in the paper rely entirely upon the robustness of this scoring.”**
  - This statement “**In one of the examples given, if the “highest level of chimerism observed” for a taxon was scored as a 1 by all undergraduate coders and as a ‘2’ by the lead author and expert, then the highest score remained as 1**.” is incorrect. As we say at the end of the mentioned paragraph (p.30): “Finally, while revising the manuscript, A.F. proof-read these scores based on the literature.”. In other words, if A.F. found that a score was incorrect in any of the taxa after reading the relevant literature, A.F. decided to keep or change the score. A.F. was the most senior expert on chimerism in the team.
- **“This approach to scoring appears slightly incompatible then with the second example/scenario described where if one of the undergraduate students were to score 1,1, 2 say and the expert coded 2, then the lead author would decide on the correct score suggesting that ultimately it is the lead author that possesses decisive knowledge in this area.”**
  - As mentioned here, “Finally, while revising the manuscript, A.F. proof-read these scores based on the literature.”, A.F., a senior expert on chimerism, and not the leading author, decided on the final score based on the literature.
- **“When describing the tumor invasiveness scoring, it is unclear whether the same scoring approach was taken (involving additional ‘coders’) when assessing these levels. This should be made clear.”**
  - Due to the ease of already having tumor invasiveness data in large across-taxa reviews (e.g. Aktipis et al. 2015), and the limited number of species for which transmissible cancers have been found, we did not recruit additional people to assess the tumor invasiveness scoring. S.E.K. set the tumor invasiveness scoring based on this literature. “S.E.K. collected tumour invasiveness data for 12 obligately multicellular taxa: vertebrata, tunicata, protostomia, placozoa, ctenophora, echinodermata, cnidaria, porifera, basidiomycota, ascomycota, embryophyta, and rhodophyta [[39,43,149]](https://paperpile.com/c/NUwUrz/bsJ0D+4SrH4+yQ2hT). “. To clarify this, in the “Tumour invasiveness” section in the methods, we have replaced ‘we’ with ‘S.E.K.’.
- **“There are inconsistencies within the text. For example, in the abstract the authors state the second set of associations is assessed across 11 terrestrial mammalian species. Figure 2 legend states 12 species although the text referring to Figure 2 (line 221) states 11 species.”**
  - “**For example, in the abstract the authors state the second set of associations is assessed across 11 terrestrial mammalian species. “ -** this is correct
  - “**Figure 2 legend states 12 species” -** this is correct
  - “**although the text referring to Figure 2 (line 221) states 11 species”** - this is also correct since we say here the word ‘analyse’: “We were only able to analyse 12 obligately multicellular taxa across the tree of life and 11 terrestrial mammalian species (Figs 1 and 3)”. In other words, we only analysed 11 of the 12 species shown in Figure 3, since we don’t have relevant malignancy or neoplasia prevalence data for humans. I’ve now rephrased the above sentence to “We were only able to perform correlations using 12 obligately multicellular taxa across the tree of life (Fig 1) and 11 out of the 12 terrestrial mammalian species shown in Figure 3 (excluding humans due to the absence of relevant malignancy and neoplasia prevalence data).”
  - To clarify, we also rephrased this section in the methods “We added malignancy prevalence data for adult humans ([https://ourworldindata.org](https://ourworldindata.org/grapher/share-of-deaths-by-cause)) in our analyses, but not neoplasia prevalence since we do not have an estimate of benign prevalence in adult humans.” to “We excluded malignancy and neoplasia prevalence data for adult humans ([https://ourworldindata.org](https://ourworldindata.org/grapher/share-of-deaths-by-cause)) from our analyses because the malignancy and neoplasia data from animals under human care are collected from necropsies whereas the malignancy data in [https://ourworldindata.org](https://ourworldindata.org/grapher/share-of-deaths-by-cause) are not collected from necropsies, and because we do not have an estimate of benign prevalence in adult humans.” adding a reason why we excluded the malignancy and neoplasia prevalence data in humans. We also rephrased this phrase in the legend “In the case of adult humans, we only show their malignancy prevalence” to “In the case of adult humans, we only show their malignancy prevalence from [https://ourworldindata.org](https://ourworldindata.org/grapher/share-of-deaths-by-cause)” for clarification.
- **“When applying PGLS, standard approaches assume a homogeneous model of evolution across the phylogenetic tree. Is that what the authors expect in considering these traits? How is this justified?”**
  - In our analyses we have relied on the precedent sent in comparative phylogenetics that the assumption of Brownian motion evolution is a fair one. Further, the central role of the PGLS regression is to control for some proportion of the variance in the trait data that is due to shared ancestry. It is assumed that whatever variance in the trait data that is not proportional to branch length has been shifted by evolution. However, the mode of evolution does not change the controlled factor in the regression.
- **Reviewer #2**

**“I think that it’s more opportune to move “Ecology may influence chimerism” paragraph under results section.”**

- Given that this paragraph also discusses our results in relation to previous literature, we think it would be more suitable in the discussion section where it is at the moment.

However, we recognise that this result has not been mentioned in the results section, therefore, we have now added this paragraph in the results section “Chimerism levels in terrestrial vs. aquatic taxa

Among the examined 12 obligately multicellular taxa, there was no significant correlation between chimerism levels and species being terrestrial vs. aquatic. In other words, obligately multicellular lineages that evolved on land did not have significantly different levels of chimerism than obligately multicellular taxa that evolved in water (S1 Fig). “

- **“The manuscript is very interesting, but I found difficult to understand some points due to the lack of definitions for people who are not experts in this field.”**
  - We provide a definition of chimerism in the introduction: “A chimera is an obligately multicellular organism that is composed of non-clonal cells (relatedness < 1), which do not originate from mutations within the body [[3–5]](https://paperpile.com/c/NUwUrz/4J1s3+gnfuK+YLAge). Chimeric cells are cells from a different host. This can range from large scale cellular exchange to smaller amounts, called microchimerism.”
  - We also explain obligate multicellularity in the introduction: “irreversible (obligate) multicellularity”
  - We also provide an explanation of the acronyms used in Table S2 in the table’s legend.
- **“Moreover, not all results are critically discussed.”**
  - We have now added this section in the results section:

“Chimerism levels in terrestrial vs. aquatic taxa

Among the examined 12 obligately multicellular taxa, there was no significant correlation between chimerism levels and species being terrestrial vs. aquatic. In other words, obligately multicellular lineages that evolved on land did not have significantly different levels of chimerism than obligately multicellular taxa that evolved in water (S1 Fig). “

- **“The manuscript is a little confused in some parts and difficult to understand. I suggest improving it by making the above concepts simpler, perhaps even giving some examples. For example, page 3: “these unaligned fitness interest…can lead to fatal cancers”. Please, give some examples.”**
  - We added this as an example just after the sentence that the reviewer is referring to. “For example, detection of Y chromosomes in the mother (male microchimerism) has been associated with a higher risk of developing colon cancer (Kamper-Jørgensen et al. 2009). However, male microchimerism has also been associated with a decreased risk of developing breast cancer (Kamper-Jørgensen et al. 2009).”
  - With these new references, therefore, we also updated the reference numbering in the manuscript.
- **“Results on page 5-6: you define tumour invasiveness based on the presence/absence of cancer growth/ growth-like or the presence of transmissible cancers. In human cancer biology, tumour invasiveness occurs when cancer cells spread in the body through circulation and lymphatic system and are able to metastasize organs different from the primary site. Can you give examples of tumour invasiveness related to other taxa?”**
  - We explain what we define as tumor invasiveness in the results section “We classified tumour invasiveness on a scale from *no cancer or no cancer-like growth*, *cancer-like growth*, *cancer*, to *transmissible cancers*.” and in the methods section “In order to create a scale of tumour invasiveness, S.E.K. classified tumour invasiveness from lowest to highest, as no cancer/cancer-like growth detected (0) (in studies that sought to find cancer/cancer-like growth), cancer-like growth (1), cancer (2), and transmissible cancer (3).”
  - We provide examples of the highest level of tumor invasiveness (i.e., transmissible cancers) in other taxa in the introduction “Cancer cells can even invade other hosts, such as humans [[33–36]](https://paperpile.com/c/NUwUrz/x2zFh+OzNld+93j8L+1jjI9), Tasmanian devils, Syrian hamsters, dogs, clams, and molluscs as intraspecies or interspecies transmissible cancers [[37–43]](https://paperpile.com/c/NUwUrz/af0zW+Zkv78+PXHXw+yQ2hT+yg93W+m8zXT+UpOt1). “ and “For instance, transmissible cancers are linked with loss of diversity in the major histocompatibility complex (MHC), low expression of MHC antigens, in other words immunological invisibility, and appear to be boundless in terms of the intraspecific hosts they can invade (e.g., 4,000-8,500 year old distribution of Canine Transmissible Venereal Tumours across 43 countries) [[50]](https://paperpile.com/c/NUwUrz/5CjsP).”
- **Please, could you explain these data in an easier way? What did you mean with higher tumour invasiveness will be observed in taxa that accept foreign cells from different species? This is not the case of humans in which microchimerism is observed between the same species.**
  - We mention the following in the results section “We found that across 12 obligately multicellular taxa, the highest level of tumour invasiveness observed in a taxon is positively correlated with the highest level of chimerism observed in a taxon. In other words, in obligately multicellular taxa that accept foreign cells from different species, higher tumour invasiveness will be observed “. The 2nd sentence here essentially explains the 1st sentence. We talk here about the correlation between “highest level of tumour invasiveness observed in a taxon” and the “highest level of chimerism observed in a taxon”. The exact values (scores) of “highest level of tumour invasiveness observed in a taxon” are shown in Figs 1 and 2, as well as in the Supporting Data. Also, in S2 Table there are many examples of species within vertebrates, including humans, with a chimerism score of 3 (S2 Table: 2nd column), which justifies why the “highest level of chimerism observed” in vertebrates is 3 (Figs 1 and 2; S1 Fig).
- **Since an important point of the discussion is related to transmissible cancers, can you explain somethings more in the results section.**
  - In the results section we mention “We classified tumour invasiveness on a scale from *no cancer or no cancer-like growth*, *cancer-like growth*, *cancer*, to *transmissible cancers*. We found that across 12 obligately multicellular taxa, the highest level of tumour invasiveness observed in a taxon is positively correlated with the highest level of chimerism observed in a taxon.” and in the same section we also mention “S.E.K. collected tumour invasiveness data for 12 obligately multicellular taxa: vertebrata, tunicata, protostomia, placozoa, ctenophora, echinodermata, cnidaria, porifera, basidiomycota, ascomycota, embryophyta, and rhodophyta [[40,44,150]](https://paperpile.com/c/NUwUrz/bsJ0D+4SrH4+yQ2hT).”. Our study basically used these references to categorise each taxon according to the highest level of tumor invasiveness observed in that taxon. We did not generate new (e.g., functional or clinical) data on transmissible cancers. Therefore, we don’t think it would be appropriate to expand more on transmissible cancers in the results section.
- **What is the difference between malignant neoplasia and malignancy in your study? Give a definition of these two terms in this context.**
  - Malignant neoplasia and malignancy have the same meaning. However, we do not mention the phrase “malignant neoplasia” anywhere in the text. We provide the following explanation for neoplasia prevalence as well as malignancy prevalence in the text. In the methods section we mention “Neoplasia includes benign or malignant tumours. Malignancy prevalence or neoplasia prevalence refers to the total malignant records of a species including non-neoplasia records or neoplasia records excluding non-neoplasia records, divided by the total records with denominators, respectively.”
- **Discussion on page 15: I suggest reporting what are the similar mechanisms that cancer and chimeric cells share.**
  - We mention the known common mechanisms that cancer and chimeric cells share in the introduction: “Cancers and chimerism share some of these hallmarks. Cancer cells can evade immune destruction [[45–48]](https://paperpile.com/c/NUwUrz/EO5uJ+VBWO8+FI5cl+quWPL); chimeric cells can too [[24,49]](https://paperpile.com/c/NUwUrz/U89ic+TTJcC). For instance, transmissible cancers are linked with loss of diversity in the major histocompatibility complex (MHC), low expression of MHC antigens, in other words immunological invisibility, and appear to be boundless in terms of the intraspecific hosts they can invade (e.g., 4,000-8,500 year old distribution of Canine Transmissible Venereal Tumours across 43 countries) [[50]](https://paperpile.com/c/NUwUrz/5CjsP). The membrane protein CD200 inhibits natural killer cell responses to cancer, facilitates graft tolerance in humans and mice, and is highly expressed on transmissible devil facial tumour cells [[51]](https://paperpile.com/c/NUwUrz/mwtkn)*.* Also, both cancer cells and chimeric endothelial progenitor fetal cells can induce angiogenesis and activate invasion [[24,44,52–55]](https://paperpile.com/c/NUwUrz/4SrH4+TTJcC+GBDYf+CNP3G+iOAts+gOsVz). Chimeric fetal cells have been found in several tumours in mothers [[56–59]](https://paperpile.com/c/NUwUrz/zAnTe+JuqcA+PXfiy+VVUru). Furthermore, chimeric fetal cells are more often found in cancerous than healthy tissue [[60–62]](https://paperpile.com/c/NUwUrz/m62dt+NBPM6+r0r1F). Genetic differences among cells can lead to conflict over limited resources, and overproliferation of one cell type over the other [[8]](https://paperpile.com/c/NUwUrz/FW5ML). This may be happening when allografts or xenografts lead to hyperplasias in sea cucumbers, sea stars, and sponges [[63–65]](https://paperpile.com/c/NUwUrz/o5Ilz+RuG3i+fmzBC), and interspecific crosses can lead to cancer in plants [[44,66]](https://paperpile.com/c/NUwUrz/W8OCr+4SrH4). “
