# Supplementary material for: Is chimerism associated with cancer across the tree of life?
Source: PLoS One. 2023 Jun 29;18(6):e0287901. doi: 10.1371/journal.pone.0287901 (PMC10309991; doi:10.1371/journal.pone.0287901)
Supplement: Peer review history

# Peer review: Decision Letter 0

**DOI:** 10.1371/journal.pone.0287901.r001

**Authors:** Michael Schubert

7 Mar 2023

PONE-D-23-01880Is chimerism associated with cancer across the tree of life?PLOS ONE

Dear Dr. Kapsetaki,

Thank you for submitting your manuscript to PLOS ONE. After careful consideration, we feel that it has merit but does not fully meet PLOS ONE’s publication criteria as it currently stands. Therefore, we invite you to submit a revised version of the manuscript that addresses the points raised during the review process.

Please submit your revised manuscript by Apr 21 2023 11:59PM. If you will need more time than this to complete your revisions, please reply to this message or contact the journal office at plosone@plos.org. When you're ready to submit your revision, log on to https://www.editorialmanager.com/pone/ and select the 'Submissions Needing Revision' folder to locate your manuscript file.

Please include the following items when submitting your revised manuscript:

- A rebuttal letter that responds to each point raised by the academic editor and reviewer(s). You should upload this letter as a separate file labeled 'Response to Reviewers'.
- A marked-up copy of your manuscript that highlights changes made to the original version. You should upload this as a separate file labeled 'Revised Manuscript with Track Changes'.
- An unmarked version of your revised paper without tracked changes. You should upload this as a separate file labeled 'Manuscript'.

If you would like to make changes to your financial disclosure, please include your updated statement in your cover letter. Guidelines for resubmitting your figure files are available below the reviewer comments at the end of this letter.

If applicable, we recommend that you deposit your laboratory protocols in protocols.io to enhance the reproducibility of your results. Protocols.io assigns your protocol its own identifier (DOI) so that it can be cited independently in the future. For instructions see: https://journals.plos.org/plosone/s/submission-guidelines#loc-laboratory-protocols. Additionally, PLOS ONE offers an option for publishing peer-reviewed Lab Protocol articles, which describe protocols hosted on protocols.io. Read more information on sharing protocols at https://plos.org/protocols?utm_medium=editorial-email&utm_source=authorletters&utm_campaign=protocols.

We look forward to receiving your revised manuscript.

Kind regards,

Michael Schubert

Academic Editor

PLOS ONE

Journal Requirements:

When submitting your revision, we need you to address these additional requirements.

1. Please ensure that your manuscript meets PLOS ONE's style requirements, including those for file naming. The PLOS ONE style templates can be found at 

https://journals.plos.org/plosone/s/file?id=wjVg/PLOSOne_formatting_sample_main_body.pdf and 

https://journals.plos.org/plosone/s/file?id=ba62/PLOSOne_formatting_sample_title_authors_affiliations.pdf

2. Thank you for stating the following financial disclosure: 

  "This work was supported in part by NIH grants U54 CA217376, U2C CA233254, P01 CA91955, and R01 CA140657 as well as CDMRP Breast Cancer Research Program Award BC132057 and the Arizona Biomedical Research Commission grant ADHS18-198847. The findings, opinions and recommendations expressed here are those of the authors and not necessarily those of the universities where the research was performed or the National Institutes of Health."

Please state what role the funders took in the study.  If the funders had no role, please state: "The funders had no role in study design, data collection and analysis, decision to publish, or preparation of the manuscript." 

If this statement is not correct you must amend it as needed. 

Please include this amended Role of Funder statement in your cover letter; we will change the online submission form on your behalf.

3. Please note that supplementary tables (should remain/ be uploaded) as separate "supporting information" files.

[Note: HTML markup is below. Please do not edit.]

Reviewers' comments:

Reviewer's Responses to Questions

**Comments to the Author**

1. Is the manuscript technically sound, and do the data support the conclusions?

The manuscript must describe a technically sound piece of scientific research with data that supports the conclusions. Experiments must have been conducted rigorously, with appropriate controls, replication, and sample sizes. The conclusions must be drawn appropriately based on the data presented. 

Reviewer #1: No

Reviewer #2: Partly

**********

2. Has the statistical analysis been performed appropriately and rigorously? 

Reviewer #1: No

Reviewer #2: I Don't Know

**********

3. Have the authors made all data underlying the findings in their manuscript fully available?

The PLOS Data policy requires authors to make all data underlying the findings described in their manuscript fully available without restriction, with rare exception (please refer to the Data Availability Statement in the manuscript PDF file). The data should be provided as part of the manuscript or its supporting information, or deposited to a public repository. For example, in addition to summary statistics, the data points behind means, medians and variance measures should be available. If there are restrictions on publicly sharing data—e.g. participant privacy or use of data from a third party—those must be specified.

Reviewer #1: No

Reviewer #2: Yes

**********

4. Is the manuscript presented in an intelligible fashion and written in standard English?

PLOS ONE does not copyedit accepted manuscripts, so the language in submitted articles must be clear, correct, and unambiguous. Any typographical or grammatical errors should be corrected at revision, so please note any specific errors here.

Reviewer #1: Yes

Reviewer #2: Yes

**********

5. Review Comments to the Author

Please use the space provided to explain your answers to the questions above. You may also include additional comments for the author, including concerns about dual publication, research ethics, or publication ethics. (Please upload your review as an attachment if it exceeds 20,000 characters)

Reviewer #1: Based on a review of available literature, this article assesses whether there is an association between chimerism and cancer across 12 obligately multicellular taxa. The authors also test for an association between chimerism and tumor invasiveness, neoplasia prevalence, and malignancy prevalence across terrestrial mammalian species.

The question is well motivated and is one of interest to the transmissible cancer and broader cancer communities and the authors present an ideal assembly of investigators for this problem. However the data and approach presented in the manuscript is weak. The authors themselves state that this analysis is an ‘imprecise view of the relationship between chimerism and cancer’ which I tend to agree with.

I question the appropriateness of the method by which the highest chimerism level was scored for taxa. No weighting is given to the amount or degree of literature support for a score, or no threshold used, within a certain taxon which might be one approach to tackle unavoidable sampling or reporting bias in a systematic literature review of this kind. No attempt appears to be made to mitigate this bias although it is acknowledged. The authors do not present decision criteria used to determine whether or not a given study should be included beyond presence or absence of key words.

In one of the examples given, if the “highest level of chimerism observed” for a taxon was scored as a 1 by all undergraduate coders and as a ‘2’ by the lead author and expert, then the highest score remained as 1. Essentially meaning that where an expert disagreed with inexpert (by virtue of exposure/experience) undergraduates working over just a 3 week period, the undergraduate score was retained. Considering the complexity of establishing a highest degree of chimerism for an entire taxon based on synthesis of the literature, I’m not at all convinced that a scenario like this wouldn’t reflect some misunderstanding on the part of the recruited undergraduates. In particular where it is clear that some of these coders do not have undergraduate training in biology (text states some of the peer-reviewers are psychology majors). There is no validation of repeatability for this scoring system and the associations being tested in the paper rely entirely upon the robustness of this scoring.

This approach to scoring appears slightly incompatible then with the second example/scenario described where if one of the undergraduate students were to score 1,1, 2 say and the expert coded 2, then the lead author would decide on the correct score suggesting that ultimately it is the lead author that possesses decisive knowledge in this area.

When describing the tumor invasiveness scoring, it is unclear whether the same scoring approach was taken (involving additional ‘coders’) when assessing these levels. This should be made clear.

There are inconsistencies within the text. For example, in the abstract the authors state the second set of associations is assessed across 11 terrestrial mammalian species. Figure 2 legend states 12 species although the text referring to Figure 2 (line 221) states 11 species.

When applying PGLS, standard approaches assume a homogeneous model of evolution across the phylogenetic tree. Is that what the authors expect in considering these traits? How is this justified?

I cannot recommend this paper for publication in its current form.

Reviewer #2: In the study of Kapsetaki and colleagues it has been reported that taxa with higher levels of chimerism have higher tumour invasiveness, though no association was observed between malignancy or neoplasia and chimerism among mammals. The Authors suggest that there may be an important biological relationship between chimerism and susceptibility to tissue invasion by tumour cells. These findings could also have implications for the identification of tumour invasion mechanisms and for the study of transmissible cancers. The manuscript is very interesting, but I found difficult to understand some points due to the lack of definitions for people who are not experts in this field. Moreover, not all results are critically discussed. The manuscript is a little confused in some parts and difficult to understand. I suggest improving it by making the above concepts simpler, perhaps even giving some examples.

For example, page 3: “these unaligned fitness interest…can lead to fatal cancers”. Please, give some examples.

Results on page 5-6: you define tumour invasiveness based on the presence/absence of cancer growth/ growth-like or the presence of transmissible cancers. In human cancer biology, tumour invasiveness occurs when cancer cells spread in the body through circulation and lymphatic system and are able to metastasize organs different from the primary site. Can you give examples of tumour invasiveness related to other taxa?

Please, could you explain these data in an easier way? What did you mean with higher tumour invasiveness will be observed in taxa that accept foreign cells from different species? This is not the case of humans in which microchimerism is observed between the same species.

Since an important point of the discussion is related to transmissible cancers, can you explain somethings more in the results section.

What is the difference between malignant neoplasia and malignancy in your study? Give a definition of these two terms in this context.

Discussion on page 15: I suggest reporting what are the similar mechanisms that cancer and chimeric cells share.

I think that it’s more opportune to move “Ecology may influence chimerism” paragraph under results section.

**********

6. PLOS authors have the option to publish the peer review history of their article (what does this mean?). If published, this will include your full peer review and any attached files.

If you choose “no”, your identity will remain anonymous but your review may still be made public.

**Do you want your identity to be public for this peer review?** For information about this choice, including consent withdrawal, please see our Privacy Policy.

Reviewer #1: No

Reviewer #2: No

**********

[NOTE: If reviewer comments were submitted as an attachment file, they will be attached to this email and accessible via the submission site. Please log into your account, locate the manuscript record, and check for the action link "View Attachments". If this link does not appear, there are no attachment files.]

While revising your submission, please upload your figure files to the Preflight Analysis and Conversion Engine (PACE) digital diagnostic tool, https://pacev2.apexcovantage.com/. PACE helps ensure that figures meet PLOS requirements. To use PACE, you must first register as a user. Registration is free. Then, login and navigate to the UPLOAD tab, where you will find detailed instructions on how to use the tool. If you encounter any issues or have any questions when using PACE, please email PLOS at figures@plos.org. Please note that Supporting Information files do not need this step.

# Author comment: Author response to Decision Letter 0

**DOI:** 10.1371/journal.pone.0287901.r002

21 Apr 2023

We provide our "Response to Reviewers" letter.

Attachment Submitted filename: Response to Reviewers.docx Click here for additional data file.

# Peer review: Decision Letter 1

**DOI:** 10.1371/journal.pone.0287901.r003

**Authors:** Michael Schubert

15 Jun 2023

Is chimerism associated with cancer across the tree of life?

PONE-D-23-01880R1

Dear Dr. Kapsetaki,

We’re pleased to inform you that your manuscript has been judged scientifically suitable for publication and will be formally accepted for publication once it meets all outstanding technical requirements.

Within one week, you’ll receive an e-mail detailing the required amendments. When these have been addressed, you’ll receive a formal acceptance letter and your manuscript will be scheduled for publication.

An invoice for payment will follow shortly after the formal acceptance. To ensure an efficient process, please log into Editorial Manager at http://www.editorialmanager.com/pone/, click the 'Update My Information' link at the top of the page, and double check that your user information is up-to-date. If you have any billing related questions, please contact our Author Billing department directly at authorbilling@plos.org.

If your institution or institutions have a press office, please notify them about your upcoming paper to help maximize its impact. If they’ll be preparing press materials, please inform our press team as soon as possible -- no later than 48 hours after receiving the formal acceptance. Your manuscript will remain under strict press embargo until 2 pm Eastern Time on the date of publication. For more information, please contact onepress@plos.org.

Kind regards,

Michael Schubert

Academic Editor

PLOS ONE

Reviewers' comments:

Reviewer's Responses to Questions

**Comments to the Author**

1. If the authors have adequately addressed your comments raised in a previous round of review and you feel that this manuscript is now acceptable for publication, you may indicate that here to bypass the “Comments to the Author” section, enter your conflict of interest statement in the “Confidential to Editor” section, and submit your "Accept" recommendation.

Reviewer #2: All comments have been addressed

**********

2. Is the manuscript technically sound, and do the data support the conclusions?

The manuscript must describe a technically sound piece of scientific research with data that supports the conclusions. Experiments must have been conducted rigorously, with appropriate controls, replication, and sample sizes. The conclusions must be drawn appropriately based on the data presented. 

Reviewer #2: Yes

**********

3. Has the statistical analysis been performed appropriately and rigorously? 

Reviewer #2: I Don't Know

**********

4. Have the authors made all data underlying the findings in their manuscript fully available?

The PLOS Data policy requires authors to make all data underlying the findings described in their manuscript fully available without restriction, with rare exception (please refer to the Data Availability Statement in the manuscript PDF file). The data should be provided as part of the manuscript or its supporting information, or deposited to a public repository. For example, in addition to summary statistics, the data points behind means, medians and variance measures should be available. If there are restrictions on publicly sharing data—e.g. participant privacy or use of data from a third party—those must be specified.

Reviewer #2: Yes

**********

5. Is the manuscript presented in an intelligible fashion and written in standard English?

PLOS ONE does not copyedit accepted manuscripts, so the language in submitted articles must be clear, correct, and unambiguous. Any typographical or grammatical errors should be corrected at revision, so please note any specific errors here.

Reviewer #2: Yes

**********

6. Review Comments to the Author

Please use the space provided to explain your answers to the questions above. You may also include additional comments for the author, including concerns about dual publication, research ethics, or publication ethics. (Please upload your review as an attachment if it exceeds 20,000 characters)

Reviewer #2: (No Response)

**********

7. PLOS authors have the option to publish the peer review history of their article (what does this mean?). If published, this will include your full peer review and any attached files.

If you choose “no”, your identity will remain anonymous but your review may still be made public.

**Do you want your identity to be public for this peer review?** For information about this choice, including consent withdrawal, please see our Privacy Policy.

Reviewer #2: No

**********
